# Structure elucidation of metabolite x17299 by interpretation of mass spectrometric data

**DOI:** 10.1007/s11306-017-1231-x

**Published:** 2017-06-24

**Authors:** Qibo Zhang, Lisa A. Ford, Anne M. Evans, Douglas R. Toal

**Affiliations:** Metabolon, Inc., 617 Davis Drive, Suite 400, Morrisville, NC 27560 USA

**Keywords:** x17299, *N,N,N*-Trimethyl-l-alanyl-l-proline betaine, Mass spectrum interpretation, Metabolite structure elucidation

## Abstract

**Introduction:**

A major bottleneck in metabolomic studies is metabolite identification from accurate mass spectrometric data. Metabolite x17299 was identified in plasma as an unknown in a metabolomic study using a compound-centric approach where the associated ion features of the compound were used to determine the true molecular mass.

**Objectives:**

The aim of this work is to elucidate the chemical structure of x17299, a new compound by de novo interpretation of mass spectrometric data.

**Methods:**

An Orbitrap Elite mass spectrometer was used for acquisition of mass spectra up to MS^4^ at high resolution. Synthetic standards of *N,N,N*-trimethyl-l-alanyl-l-proline betaine (l,l-TMAP), a diastereomer, and an enantiomer were chemically prepared.

**Results:**

The planar structure of x17299 was successfully proposed by de novo mechanistic interpretation of mass spectrometric data without any laborious purification and nuclear magnetic resonance spectroscopic analysis. The proposed structure was verified by deuterium exchanged mass spectrometric analysis and confirmed by comparison to a synthetic standard. Relative configuration of x17299 was determined by direct chromatographic comparison to a pair of synthetic diastereomers. Absolute configuration was assigned after derivatization of x17299 with a chiral auxiliary group followed by its chromatographic comparison to a pair of synthetic standards.

**Conclusion:**

The chemical structure of metabolite x17299 was determined to be l,l-TMAP.

**Electronic supplementary material:**

The online version of this article (doi:10.1007/s11306-017-1231-x) contains supplementary material, which is available to authorized users.

## Introduction

Nuclear magnetic resonance (NMR) spectra can provide excellent information about the chemical structures of organic compounds, and hence structural elucidation of new compounds relies mainly on the interpretation of NMR spectra. The development of high field magnet, new pulse programs, liquid chromatography coupled NMR, as well as capillary and cryogenic probes has made NMR spectroscopy much more sensitive and effective (Gonnella 2012; Battistel et al. 2014; Gökay and Albert 2012). Unfortunately, NMR experiments generally require a purified sample in sufficient quantity, which often demands laborious and challenging efforts for any minor component in a complex mixture.

Mass spectrometry is another powerful technique that can provide important structural information upon fragmentation of the parent ions and has many applications in structure elucidation of small molecules (Kind and Fiehn 2010). Liquid chromatography coupled mass spectrometry (LC/MS) is much more sensitive and convenient than NMR and has been extensively used for identification and characterization of metabolites and degradation products in pharmaceutical and agrochemical studies (Emwas 2015; Wen and Zhu 2015; Harir et al. 2013). Because most metabolites and degradation products structurally resemble the parent compound, fragmentation information obtained from the parent compound usually facilitates the structural characterization of these related unknowns.

In untargeted metabolomic studies, identification of unknown endogenous metabolites using LC/MS is much more difficult because the parent compounds themselves are also unknown (Dias et al. 2016). Consequently, structure characterization typically ends, regardless of the result, after searching available mass spectral libraries of known compounds and rarely continues with purification and NMR analysis (Wishart 2011; Bingol et al. 2016; Watson 2013). Some software products have recently been developed to predict fragmentation spectra from molecular structures and to streamline the search process, but to our knowledge, little success has been achieved to elucidate true unknown structures solely from mass spectra using software programs (Hufsky and Böcker 2016; Tsugawa et al. 2016; Knolhoff et al. 2016; Treutler et al. 2016; Ma et al. 2014; Kind et al. 2014). LC/MS has also been widely applied to the structure characterization of large molecules, such as proteins, glycans, and antibody-drug conjugates (Aebersold and Mann 2016; Leymarie and Zaia 2012; Beck et al. 2016). The fragmentation of large molecules in a mass spectrometer often follows certain distinct pathways because large molecules are assembled with a limited number of repetitive known monomers.

Although mass spectrometry has historically played a significant role in the structural elucidation of small compounds, especially before the availability of NMR spectroscopy (Biemann 2015; Wright and Warren 1967), only a small number of structures of natural products have been deduced solely by mass spectrometry and later confirmed by chemical synthesis (Dickschat 2014; Schiestl et al. 2003). As shown in textbooks, a fragmentation spectrum usually contains abundant structural information that can be harnessed for structural elucidation of the example compound. In reality, however, interpretation of mass spectra of an unknown compound for a successful structural proposal remains as a formidable task, perhaps due to inadequate understanding of the complex fragmentation chemistry in gas phase and the natural limitation of MS technology itself that does not necessarily provide a comparable level of atom connectivity as NMR. Consequently, the current application of mass spectrometry to the structural elucidation of new compounds is mostly confined to providing molecular formula and to resolving specific issues at a later stage when most of the structural information has become available from other analysis (Lan et al. 2016; Gleye et al. 1997).

Here we report the structure elucidation of a new compound called x17299 by de novo interpretation of mass spectrometric data in the absence of any NMR analysis, which was unfeasible given the small amount of material present in the complex matrix of human plasma. The deduced structure was subsequently confirmed and stereochemistry determined by means of chemical synthesis. This compound was discovered as a potential biomarker for glomerular filtration rate (GFR) assessment after LC/MS analysis of a large number of plasma and serum samples from healthy and diseased individuals in an extensive metabolomic study aiming to more conveniently and accurately assess GFR.

## Materials and methods

### Materials

Silver oxide, anhydrous sodium sulfate, iodomethane, iodomethane-*d*_3_, l-phenylalanine methyl ester hydrochloride, mass spectrometric grade formic acid (~98%), and HPLC grade trifluoroacetic acid (99.0%) were obtained from Sigma-Aldrich; HPLC grade methanol, acetonitrile, ethyl acetate, and water from Fisher Scientific; Deuterium oxide (99.8%) and HPLC grade dichloromethane from Acros; l-alanyl-l-proline, *N*-(*tert*-butoxycarbonyl)-d-alanine, l-proline *tert*-butyl ester hydrochloride, d-proline *tert*-butyl ester hydrochloride, and 1-(3-dimethylaminopropyl)-3-ethylcarbodiimide (EDC) hydrochloride from Tokyo Chemical Industry. A Fisher Scientific vortex mixer was used for mixing and a Sorvall Legend Micro 21R microcentrifuge used for centrifugation of 1.5 mL Eppendorf tubes. A Corning Laboratory stirrer was used for mixing chemical reactions. Human plasma (Na_2_-EDTA) was obtained from Bioreclamation and stored at −80 °C. An Argonaut SPE DRY™ 96 DUAL evaporator was used for solvent evaporation.

### Chromatography

A Waters Acquity UPLC system equipped with a binary solvent manager, a refrigerated sample manager (set at 12 °C), and a column manager (set at 40 °C) was used for liquid chromatography with a reversed phase column (Waters ACQUITY UPLC^®^ BEH C18, 1.7 µm, 2.1 × 100 mm). Mobile phase A was 0.1% formic acid in water (or 0.1% formic acid in deuterium oxide for deuterium exchange experiments) and mobile phase B was 0.1% formic acid in methanol. Linear gradient elution was carried out with an initial condition of 0% mobile phase B, which was held for 2.00 min. Mobile phase B was then increased to 98% in 0.50 min and maintained for 0.90 min. Mobile phase B reverted to 0% in 0.10 min for equilibration for next injection. The flow rate was 350 µL/min and the total run time was 4.50 min. A loop fixed aliquot of 5.0 µL of the final sample solution was injected for each sample. The eluent was directly introduced into the electrospray source of a mass spectrometer. Strong needle wash was neat methanol and weak needle wash was a mixture of methanol and water (0.5:99.5). Seal wash was a mixture of methanol and water (10:90).

For chromatography of the coupling products with L-phenylalanine methyl ester, the same above liquid chromatography conditions were used except the gradient. In this case, the elution was carried out with an initial condition of 30% mobile phase B, which was increased to 40% in 2.00 min. The proportion of mobile phase B was then increased to 98% in 0.50 min and maintained for 0.90 min. Mobile phase B reverted to 30% in 0.10 min for equilibration for next injection.

### Mass spectrometry

A Thermo Scientific Orbitrap Elite mass spectrometer equipped with a heated electrospray ionization (HESI-II) probe was used in positive mode for this study. The instrument was controlled by Orbitrap Elite™ 2.7 and XCalibur™ 2.2 software. The heater temperature was set at 430 °C, sheath gas flow rate at 30, auxiliary gas flow rate at 12, sweep gas flow rate at 0, ion spray voltage at 4.20 kV, capillary temperature at 350 °C, and S-lens RF level at 65%. A resolution of 30,000 was used to collect full scan FTMS spectra with mass range between *m/z* 100 and 300. For all MS fragmentation experiments, a resolution of 15,000 was used along with activation Q of 0.250 and activation time of 10.0 ms. The normalized collision energy for MS^2^ experiment was 31.0 eV with an isolation width of 1.0 *m/z* and scan range between *m/z* 60 and 240. For the MS^3^ experiment of *m/z* 229.15/142.09 (or 230.16/143.09 for deuterium exchange), normalized collision energy was 31.0 and 25.0 eV for first and second stage fragmentation, respectively, with isolation width of *m/z* 2.0 for both stages and scan range between *m/z* 50 and 240. For the MS^3^ experiment of *m/z* 229.15/170.08 (or 230.16/171.09 for deuterium exchange), normalized collision energy was 31.0 and 30.0 eV for first and second stage fragmentation, respectively. The isolation width was *m/z* 3.0 and 2.0 for first and second stage fragmentation, respectively, and scan range between *m/z* 50 and 240. For the MS^4^ experiment of *m/z* 229.15/142.09/114.09 (or 230.16/143.09/115.10 for deuterium exchange), normalized collision energy was 31.0, 20.0, and 20.0 eV for first to third stage fragmentation. Isolation width was *m/z* 2.0 for all the three stages and scan range between *m/z* 50 and 240. For acquisition of MS^2^ spectra of alanyl-prolines, normalized collision energy was set at 20 eV. All mass spectrometric data were acquired and processed without any lock mass and the external mass calibration was used.

For the coupling products with L-phenylalanine methyl ester, the same above MS conditions were used except the following. Full scan FTMS spectra were collected with mass range between *m/z* 200 and 450. The normalized collision energy for MS^2^ experiment was 28.0 eV with an isolation width of 1.0 *m/z* and scan range between *m/z* 105 and 400. For all MS^3^ experiments, normalized collision energy was 28.0 and 25.0 eV for first and second stage fragmentation, respectively, with isolation width of *m/z* 2.0 for both stages. Scan range was between *m/z* 90 and 340 for MS^3^ experiment of *m/z* 390.24/331.17, between *m/z* 70 and 340 for MS^3^ experiment of *m/z* 390.24/271.14, between *m/z* 65 and 340 for MS^3^ experiment of *m/z* 390.24/243.15, between *m/z* 80 and 340 for MS^3^ experiment of *m/z* 390.24/303.17, between *m/z* 50 and 340 for MS^3^ experiment of *m/z* 390.24/168.09, and between *m/z* 50 and 340 for MS^3^ experiment of *m/z* 390.24/142.09.

### Sample preparation

In a 1.5 mL Eppendorf tube were placed 100 µL of human plasma (thawed on ice) and 500 µL of methanol. The mixture was vortexed for 2 min and centrifuged at room temperature for 5 min at 14,000 rpm. The supernatant (500 µL) was transferred to a well of a 96-well plate and dried under a gentle stream of nitrogen at 40 °C. To the residue was added 200 µL of water with 0.1% formic acid, and the mixture was vortexed for 1 min, transferred to a 1.5 mL Eppendorf tube and centrifuged at room temperature for 10 min at 14,000 rpm. The supernatant was then transferred to a sample vial for LC/MS analysis.

### Chemical synthesis

#### Synthesis of *N,N,N*-trimethyl-l-alanyl-l-proline betaine (1c)

In a 4 mL glass vial with a magnetic stir bar were placed l-alanyl-l-proline (**13c**, 20.0 mg, 0.108 mmole), silver oxide (100 mg, 0.432 mmole), and 1.0 mL of methanol/water (4:1). The mixture was stirred on a magnetic stirrer at room temperature and 75 µL of iodomethane (171 mg, 1.2 mmole) added (Aurelio et al. 2004; Naresh Chary et al. 2012). The vial was loosely capped and the mixture stirred overnight at room temperature in the dark. The resulting mixture was evaporated to dryness under a gentle stream of nitrogen at 40 °C. Water (1.0 mL) was added to the residue and the mixture was sonicated for 2 min. The mixture was then transferred to a 1.5 mL Eppendorf tube and centrifuged at room temperature for 10 min at 14,000 rpm. An aliquot of the clear supernatant was diluted 10,000 fold with 0.1% formic acid in water and transferred to a sample vial for LC/MS analysis. HRESIMS *m/z* 229.1542 [M + H]^+^ (calcd for C_11_H_21_N_2_O_3_^+^, 229.1547). MS^2^ *m/z* 170.0814, 142.0864, 126.0915, 124.0758, 114.0914, 114.0550, 70.0651. MS^3^ (170.08) *m/z* 142.0862, 114.0549. MS^3^ (142.09) *m/z* 114.0913, 96.0806, 70.0650. MS^4^ (142.09/114.09) *m/z* 70.0650.

#### Synthesis of *N,N,N*-trimethyl-*d*_9_-l-alanyl-l-proline betaine

The same procedure for synthesis of **1c** was used except that iodomethane-*d*_3_ replaced iodomethane. HRESIMS *m/z* 238.2106 [M + H]^+^ (calcd for C_11_H_12_D_9_N_2_O_3_^+^, 238.2112). MS^2^ *m/z* 170.0811, 142.0862, 126.0912, 124.0756, 114.0911, 114.0548, 70.0650.

#### Synthesis of d-alanyl-l-proline (**13a**)

In a 4 mL glass vial with a magnetic stir bar were placed *N*-(*tert*-butoxycarbonyl)-d-alanine (**10**, 18.9 mg, 0.100 mmole) and l-proline *tert*-butyl ester hydrochloride (**11a**, 20.8 mg, 0.100 mmole), EDC hydrochloride (80 mg, 0.417 mmole), and acetonitrile (2 mL) (Sheehan et al. 1961). The mixture was stirred at room temperature overnight. It became a clear solution after about 1 h. The solvent was removed under a gentle stream of nitrogen at 30 °C, and the residue was partitioned between ethyl acetate and water (1 mL each). The organic phase was separated and aqueous phase extracted with ethyl acetate (2 × 1 mL). The combined organic phase was washed with water (1 mL), dried (Na_2_SO_4_), and evaporated to dryness under a stream of nitrogen at 30 °C. The residue was dissolved in a mixture of dichloromethane and trifluoroacetic acid (1 mL each). After overnight at room temperature, the solvent was removed under a stream of nitrogen at 30 °C. Acetonitrile (1 mL) was added to the residue, and then evaporated to dryness again after vortexing. A portion of the residue was dissolved in 0.1% formic acid in water and analyzed by LC/MS. The expected product eluted at 2.24 min (while l-alanyl-l-proline eluted at 1.50 min) under the same chromatographic conditions for x17299. HRESIMS *m/z* 187.1072 [M + H]^+^ (calcd for C_8_H_15_N_2_O_3_^+^, 187.1077). MS^2^ *m/z* 170.0813, 169.0973, 141.1024, 116.0706, 70.0650.

#### Synthesis of *N,N,N*-trimethyl-d-alanyl-l-proline betaine (**1a**)

The above d-alanyl-l-proline (**13a**) product residue was dissolved in 1.0 mL of methanol/water (4:1) and silver oxide (100 mg, 0.432 mmole) added along with a stir bar. The mixture was stirred on a magnetic stirrer at room temperature and 75 µL of iodomethane (171 mg, 1.2 mmole) added. The vial was loosely capped and the mixture stirred overnight at room temperature in the dark. The resulting mixture was evaporated to dryness under a gentle stream of nitrogen at 40 °C. Water (1.0 mL) was added to the residue and the mixture was sonicated for 2 min. The mixture was then transferred to a 1.5 mL Eppendorf tube and centrifuged at room temperature for 10 min at 14,000 rpm. An aliquot of the clear supernatant was diluted with 0.1% formic acid in water and analyzed by LC/MS. The expected product eluted at 2.34 min. HRESIMS *m/z* 229.1543 [M + H]^+^ (calcd for C_11_H_21_N_2_O_3_^+^, 229.1547). MS^2^ *m/z* 170.0813, 142.0864, 126.0914, 124.0757, 114.0914, 114.0550, 70.0651. MS^3^ (170.08) *m/z* 142.0863, 124.0758, 114.0551. MS^3^ (142.09) *m/z* 114.0915, 96.0806, 70.0651.

#### Synthesis of d-alanyl-d-proline (**13b**)

The same procedure for **13a** synthesis was used but starting instead with d-proline *tert*-butyl ester hydrochloride (**11b**, 20.8 mg, 0.100 mmole). The expected product eluted at 1.50 min. HRESIMS *m/z* 187.1073 [M + H]^+^ (calcd for C_8_H_15_N_2_O_3_^+^, 187.1073). MS^2^ *m/z* 170.0813, 169.0973, 141.1023, 116.0706, 70.0650.

#### Synthesis of *N,N,N*-trimethyl-d-alanyl-d-proline betaine (**1b**)

The same procedure for **1a** synthesis was used but starting instead with the d-alanyl-d-proline (**13b**) product residue. The expected product eluted around 1.41 min. HRESIMS *m/z* 229.1545 [M + H]^+^ (calcd for C_11_H_21_N_2_O_3_^+^, 229.1547). MS^2^ *m/z* 170.0814, 142.0865, 126.0915, 124.0758, 114.0916, 114.0550, 70.0651. MS^3^ (170.08) *m/z* 142.0866, 114.0553. MS^3^ (142.09) *m/z* 114.0916, 96.0809, 70.0652.

#### Synthesis of *N,N,N*-trimethyl-l-alanyl-l-prolyl-l-phenylalanine methyl ester (**15c**)

In a 4 mL glass vial were placed 100 µL of the reaction product mixture of **1c**, 100 µL of l-phenylalanine methyl ester hydrochloride (**14**, 10 mg/mL in water), 150 µL of EDC hydrochloride (10 mg/mL in water), and a stir bar (Sheehan et al. 1961). The mixture was stirred on a magnetic stirrer at room temperature overnight. An aliquot of the clear supernatant was diluted with 0.1% formic acid in a sample vial for LC/MS analysis. The expected product eluted at 1.78 min. HRESIMS *m/z* 390.2389 [M + H]^+^ (calcd for C_21_H_32_N_3_O_4_^+^, 390.2387). MS^2^ *m/z* 331.1654, 303.1703, 271.1443, 259.1443, 243.1494, 232.1336, 206.1174, 168.0894, 152.0706, 142.0864, 124.0758. MS^3^ (331.17) *m/z* 299.1396, 287.1395, 271.1446, 259.1445, 243.1497, 232.1337, 206.1180, 168.0897, 152.0709, 142.0865, 124.0759. MS^3^ (271.14) *m/z* 243.1496, 202.0867, 146.0966. MS^3^ (243.15) *m/z* 146.0967. MS^3^ (303.17) *m/z* 243.1497, 234.1127, 206.1179, 96.0811. MS^3^ (168.09) *m/z* 140.0945, 113.0711, 97.0758. MS^3^ (142.09) *m/z* 70.0652.

#### Synthesis of *N,N,N*-trimethyl-d-alanyl-d-prolyl-l-phenylalanine methyl ester (**15b**)

The same procedure for **15c** synthesis was used but starting instead with the reaction product mixture of **1b**. The expected product eluted at 2.01 min. HRESIMS *m/z* 390.2392 [M + H]^+^ (calcd for C_21_H_32_N_3_O_4_^+^, 390.2387). MS^2^ *m/z* 331.1659, 303.1709, 271.1447, 259.1447, 243.1496, 232.1339, 206.1181, 168.0897, 152.0711, 142.0866, 124.0760. MS^3^ (331.17) *m/z* 299.1395, 287.1401, 271.1446, 259.1444, 243.1495, 232.1337, 206.1179, 168.0897, 152.0708, 142.0866, 124.0759. MS^3^ (271.14) *m/z* 243.1496, 202.0869, 146.0968. MS^3^ (243.15) *m/z* 146.0968. MS^3^ (303.17) *m/z* 206.1180. MS^3^ (168.09) *m/z* 140.0943, 113.0710. MS^3^ (142.09) *m/z* 70.0652.

#### Synthesis of x17299-l-phenylalanine methyl ester

In this case, the dried plasma methanol extract in eight wells was sequentially reconstituted with aqueous 0.1 N HCl (200 µL). This concentrated extract (pH ~4.5) was centrifuged at room temperature for 10 min at 14,000 rpm and the resulting supernatant used for the derivatization. The reaction mixture in a 4 mL glass vial contained 100 µL of the concentrated extract, 100 µL of **14** (50 mg/mL in water), and 150 µL of EDC hydrochloride (50 mg/mL in water). The mixture was kept at room temperature overnight and an aliquot was diluted 2.5 fold with water containing 0.1% formic acid in a sample vial for LC/MS analysis. The expected product eluted at 1.78 min. HRESIMS *m/z* 390.2398 [M + H]^+^ (calcd for C_21_H_32_N_3_O_4_^+^_,_ 390.2387). MS^2^ *m/z* 331.1650, 303.1699, 271.1440, 259.1435, 243.1491, 232.1334, 206.1171, 168.0891, 152.0703, 142.0862, 124.0756. MS^3^ (331.17) *m/z* 299.1399, 287.1408, 271.1447, 259.1444, 243.1496, 232.1336, 206.1180, 168.0897, 152.0707, 142.0866, 124.0757. MS^3^ (271.14) *m/z* 243.1497, 202.0881, 146.0968. MS^3^ (243.15) *m/z* 146.0968.

In another experiment, an aqueous l,l-TMAP-*d*_9_ solution was spiked into the concentrated plasma extract to give a concentration comparable to that of x17299. This spiked plasma extract was treated with the same coupling procedure. The expected *d*_9_-labeled coupling product co-eluted with x17299-l-phenylalanine methyl ester. HRESIMS *m/z* 399.2947 [M + H]^+^ (calcd for C_21_H_23_D_9_N_3_O_4_^+^_,_ 399.2952). MS^2^ *m/z* 331.1653, 303.1703, 271.1442, 259.1440, 243.1493, 232.1334, 206.1177, 168.0895, 152.0707, 142.0864, 124.0756.

## Results and discussion

**Fig. 1:**
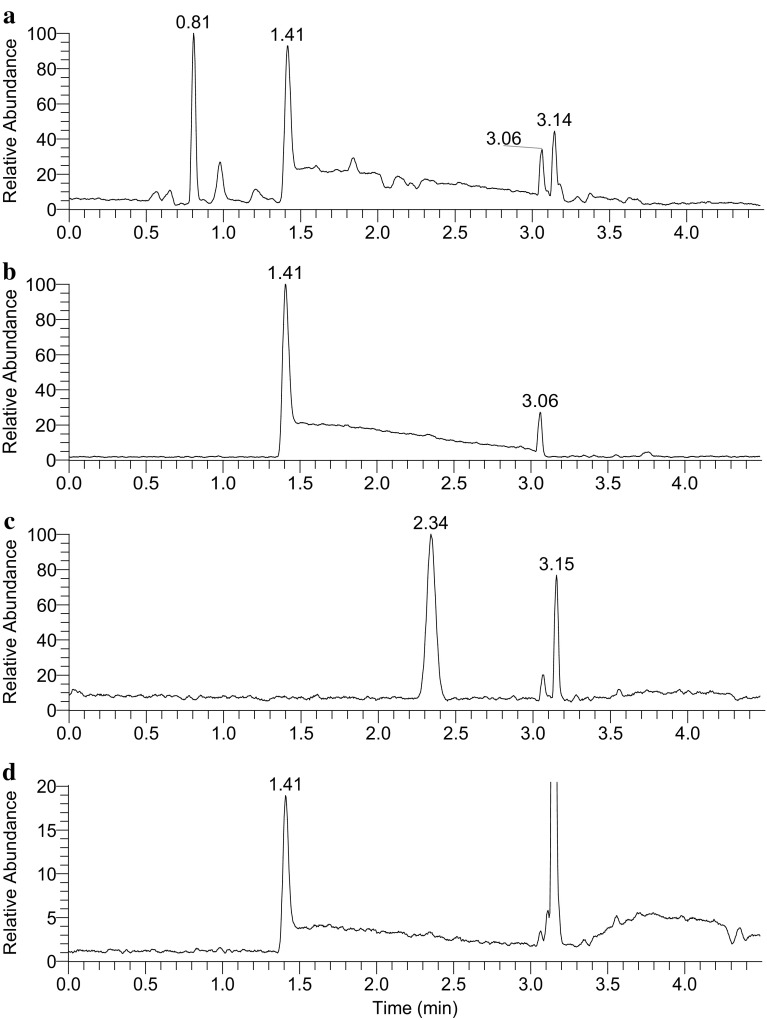
LC-MS/MS chromatograms of x17299 (1.41 min, **a**) in a plasma extract, synthetic l,l-TMAP (1.41 min, **b**), d,l-TMAP (2.34 min, **c**), and d,d-TMAP (1.41 min, **d**)

**Fig. 2:**
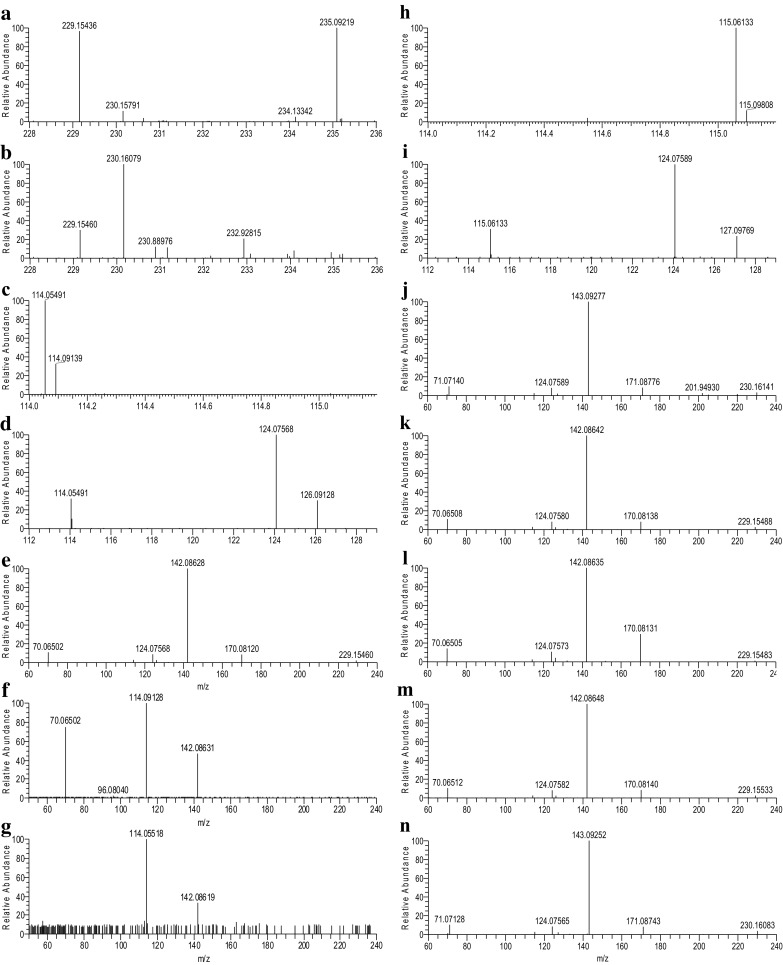
Expanded MS spectra of x17299 (**a**) and deuterated x17299 (**b**), MS^2^ spectra of x17299 (**e**) and deuterated x17299 (**j**) with corresponding expansions (**c, d, h, i**), MS^3^ spectra of *m/z* 229/142 (**f**) and 229/170 (**g**) of x17299 in a plasma extract, and MS^2^ spectra of synthetic l,l-TMAP (**k**), d,l-TMAP (**l**), d,d-TMAP (**m**), and deuterated l,l-TMAP (**n**)

An Orbitrap Elite mass spectrometer was used in this study for acquisition of mass spectra at high resolution. All measured masses are within 5 ppm of theoretical calculations of the proposed ions. Human plasma extract was chromatographed under reversed phase conditions using a UPLC system. The very polar x17299 eluted around 1.4 min with a noticeable tailing peak shape at 100% aqueous mobile phase (Fig. 1a). No effort was made to improve the chromatographic peak shape, which happened to be valuable later for its comparison to synthetic standards. One challenge associated with structure elucidation of an unknown compound in metabolomic studies is the fact that any detected ion feature can simply be a chemical adduct, multimer, or in-source fragment of a given compound. In this case, taking a compound-centric approach, where cross sample correlation was used to analyze all the ion features of a specific compound (DeHaven et al. 2010), allowed correct identification of the molecular mass. The ion features associated with x17299 did not match any compound in an in-house spectral library of over 4000 authentic standards (Evans et al. 2009, 2014). The monoisotopic mass of the protonated x17299 was determined to be 229.1544 (Fig. 2a), which supports a chemical formula of C_11_H_21_O_3_N_2_^+^ with only −1.4 ppm off the calculated mass of 229.1548 (Fig. S1). The double bond equivalent (DBE) was calculated to be 3 for the neutral compound. Collision induced dissociation (CID) of the protonated molecule produced seven daughter ions (Tables 1, S1; Fig. 2c–e), which are *m/z* 170, 142, 126, 124, 114.09, 114.05, and 70 (all collected with accurate mass, but omitted for simplicity). Further CID fragmentation of the predominant *m/z* 142 ion generated *m/z* 114.09, 70, as well as an *m/z* 96 ion (Fig. 2f), which was not detected in the MS^2^ spectrum. The *m/z* 114.05 ion was not detected by fragmentation of *m/z* 142, but instead it was generated along with the m/z 142 ion by fragmentation of *m/z* 170 (Fig. 2g). The *m/z* 70 ion was detected when *m/z* 114.09 was further fragmented (MS^4^, Fig. S2). While the molecular formula matched tens of thousands of structures in the literature, no compounds could obviously produce the same fragments generated by x17299.

**Table 1:** Calculated and measured mass of detected ions

Ion	Formula	Calculated	x17299		l,l-TMAP		d,l-TMAP		d,d-TMAP	
			Measured	Bias^a^	Measured	Bias^a^	Measured	Bias^a^	Measured	Bias^a^
**1**	C_11_H_21_N_2_O_3_^+^	229.1547	229.1544	−1.4	229.1542	−2.3	229.1543	−1.5	229.1545	−0.8
**1d**	C_11_H_20_DN_2_O_3_^+^	230.1610	230.1608	−0.7	230.1603	−2.7	n		n	
**2**	C_8_H_12_NO_3_^+^	170.0812	170.0812	0.2	170.0814	1.2	170.0813	0.8	170.0814	1.4
**2d**	C_8_H_11_DNO_3_^+^	171.0875	171.0878	1.8	171.0874	−0.1	n		n	
**3**	C_7_H_12_NO_2_^+^	142.0863	142.0863	0.1	142.0864	1.1	142.0864	0.6	142.0865	1.5
**3d**	C_7_H_11_DNO_2_^+^	143.0925	143.0928	1.7	143.0925	−0.1	n		n	
**4**	C_7_H_12_NO^+^	126.0913	126.0913	−0.5	126.0915	1.0	126.0914	0.4	126.0915	1.3
**4d**	C_7_H_11_DNO^+^	127.0976	127.0977	0.6	127.0976	−0.1	n		n	
**5**	C_7_H_10_NO^+^	124.0757	124.0757	−0.1	124.0758	0.9	124.0757	0.3	124.0758	1.0
**6**	C_6_H_12_NO^+^	114.0913	114.0914	0.4	114.0914	0.9	114.0914	0.3	114.0916	1.8
**6d**	C_6_H_11_DNO^+^	115.0976	115.0981	4.0	115.0975	−1.0	n		n	
**7**	C_5_H_8_NO_2_^+^	114.0550	114.0549	−0.4	114.0550	0.7	114.0550	0.0	114.0550	0.1
**7d**	C_5_H_7_DNO_2_^+^	115.0612	115.0613	0.9	115.0612	−0.4	n		n	
**8**	C_6_H_10_N^+^	96.0808	96.0804	−4.0	96.0806	−1.5	96.0806	−2.0	96.0809	1.4
**9**	C_4_H_8_N^+^	70.0651	70.0650	−1.6	70.0651	−0.7	70.0651	−1.1	70.0651	−0.1
**9d**	C_4_H_7_DN^+^	71.0714	71.0714	0.0	71.0713	−1.7	n		n	

*n* not measured

^a^In ppm

### Structure proposal by interpretation of mass spectrometric data

The formation of the *m/z* 170 daughter ion can be rationalized by neutral loss of a saturated amine (C_3_H_9_N) from the parent ion. As *n*-propyl and isopropyl amino moieties are rarely found in natural products (Bick and Hai 1981; Oku et al. 2003), trimethylamine appears to be a promising candidate for the lost fragment. The *m/z* 96 ion detected in MS^3^ of the *m/z* 142 ion appears to be from the loss of a formic acid molecule, and the absence of *m/z* 124 and 126 in the same MS^3^ spectrum implicates that they may be generated directly from *m/z* 170 by loss of formic acid and carbon dioxide, respectively. These results strongly suggest the presence of a carboxylic acid group in the parent. Formation of the *m/z* 142 from *m/z* 170 is consistent with the loss of a carbon monoxide molecule. A second loss of carbon monoxide from *m/z* 142 generates *m/z* 114.09, indicating that x17299 likely contains a second carbonyl group in addition to the one in the carboxylic acid group. The only heteroatom that has not been attributed is a nitrogen, which is in the *m/z* 70 ion generated likely by the loss of an acetaldehyde molecule from *m/z* 114.09. The *m/z* 70 ion has a formula of C_4_H_8_N^+^, whose neutral species possesses 2 DBE and thus a possible ring structure. The *m/z* 114.05 fragment from *m/z* 170 appears to contain an additional CO_2_ in comparison to the *m/z* 70 ion. The presence of a nitrogen atom and a likely carboxylate group in the *m/z* 114.05 ion and the biological origin of x17299 make proline moiety a promising precursor for the *m/z* 114.05 and 70 fragments. The likely trimethylamine moiety, a carbonyl group, and the unaccounted two carbon and four hydrogen atoms may reasonably assemble an *N,N,N*-trimethyl-α-alanyl or *N,N,N*-trimethyl-β-alanyl group in a biochemical. Therefore, a possible structure of the protonated x17299 is inferred to be *N,N,N*-trimethyl-α-alanylproline (**1**). Its neutral form should be called *N,N,N*-trimethyl-α-alanylproline betaine (TMAP). The polarity of this proposed structure appears to be consistent with the observed poor chromatographic retention of x17299 under reversed phase conditions.

**Scheme 1:**
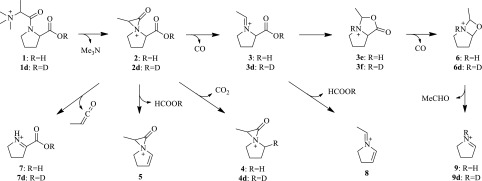
Proposed fragmentation pathways of x17299

Possible fragmentation pathways of protonated TMAP (**1**) are proposed as shown in Scheme 1. The loss of trimethylamine may be facilitated by the formation of α-lactam **2**, which loses a carbon monoxide molecule to generate the predominant *m/z* 142 ion (**3**). A β-lactam species generated from the possible β-alanyl isomer should be more stable, and loss of carbon monoxide may not be dominated and hence the β-isomer is less favorable. The ion species **3** loses another carbon monoxide, possibly via **3e**, to generate fragment **6**, which forms **9** after loss of acetaldehyde. Loss of CO_2_ and formic acid from **2** could yield **4** and **5**, respectively. Fragment **7** may be generated from **2** by loss of methylketene.

### Structure verification by deuterium exchange

The proposed structure for x17299 was first verified by a deuterium exchange experiment. Briefly, the mobile phase of chromatography was changed to deuterated solvent and the plasma extract was analyzed again. Full scan mass spectrum was acquired (Fig. S3) and a new *m/z* 230.1608 ion (Fig. 2b, −0.7 ppm off the calculated value for C_11_H_20_DN_2_O_3_^+^) detected as the major species of deuterated x17299 (**1d**), consistent with a single exchangeable proton of the proposed structure. Product ion spectrum of the *m/z* 230 ion (Fig. 2h–j; Table S2) and an MS^3^ spectrum of the *m/z* 143 ion were collected (Fig. S4). The corresponding deuterated fragments were detected at 171 (**2d**), 143 (**3d**), 127 (**4d**), 115.09 (**6d**), 115.06 (**7d**), and 71 (**9d**) (Table 1), while the fragments of 124 and 96 remained unchanged without incorporated deuterium. All these ions can be adequately rationalized into the originally proposed fragmentation pathways (Scheme 1), providing convincing evidence for validity of the proposed structure of x17299.

### Structure confirmation by comparison to a synthetic standard

**Scheme 2:**
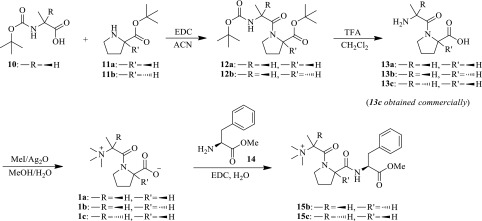
Chemical synthesis of standard compounds

Commercially available l-alanyl-l-proline (**13c**) was trimethylated by iodomethane in the presence of silver oxide to yield *N,N,N*-trimethyl-l-alanyl-l-proline betaine (l,l-TMAP, **1c**) as shown in Scheme 2. Under the same chromatographic conditions, synthetic l,l-TMAP eluted at the same retention time as x17299 with a tailing peak shape that matches that of x17299 (Fig. 1b). A predominant protonated ion of *m/z* 229.1542 (−2.3 ppm off calculated value) was detected by LC/MS (Table 1; Fig. S5), consistent with the expected reaction product. When l,l-TMAP was spiked into a plasma extract, it chromatographically co-eluted with x17299 (Fig. S6). Product ion spectrum (Fig. 2k; Table S3) of the synthetic l,l-TMAP agreed remarkably well with that of x17299 by fragments and their relative intensity (Fig. S7). Further fragmentation of the 142 and 170 daughter ions of the synthetic l,l-TMAP produced MS^3^ spectra that were identical to those from x17299 (Figs. S8, S9). An MS^4^ spectrum of the synthetic l,l-TMAP on the *m/z* 114.09 ion showed an *m/z* 70 fragment, also consistent with that of x17299 (Fig. S10). Furthermore, the synthetic l,l-TMAP was analyzed under deuterium exchange conditions and the resulting MS (Fig. S11), MS^2^ (Fig. 2n; Table S4), and MS^3^ spectra also matched those of x17299 very well (Table 1; Figs. S12, S13). All these chromatographic and mass spectrometric data support a conclusion that x17299 and TMAP share the same planar structure.

### Relative configuration determination

The structure of x17299 contains two stereogenic centers. The relative configuration of x17299 was determined by its direct comparison to *N,N,N*-trimethyl-d-alanyl-l-proline betaine (d,l-TMAP, **1a**), which was chemically synthesized from protected d-alanine and l-proline (Scheme 2). Briefly, BOC protected d-alanine (**10**) was coupled to l-proline *t*-butyl ester (**11a**) to give **12a** as promoted by EDC. After removal of the protecting groups, the dipeptide (**13a**) was characterized by mass spectrometry and by comparison to commercially available **13c** (Figs. S14, S15, S16, S17, S18). Trimethylation of **13a** yielded **1a**, which was characterized by high resolution mass spectrometry (Fig. S19) with a protonated ion of *m/z* 229.1543 (−1.5 ppm off calculated value for C_11_H_21_N_2_O_3_^+^). The product ion (Fig. 2l; Table S5) and MS^3^ spectra of d,l-TMAP were similar to those of l,l-TMAP (Figs. S20, S21, S22). All detected ions (Table 1) can be found in the fragmentation pathways (Scheme 1). However, the intensity distribution among the corresponding ions was notably different from that of l,l-TMAP. d,l-TMAP also distinguished itself by the presence of an *m/z* 124 ion in the MS^3^ spectrum of *m/z* 170 (Fig. S22), which supports the rationalized loss of formic acid from *m/z* 170. d,l-TMAP was found to elute later at 2.34 min with a much different peak shape under the same chromatographic conditions (Fig. 1c), and well separated from x17299 in a co-injected sample (Fig. S23). These results proved that d,l-TMAP and x17299 do not share the same relative configuration and that the configuration of x17299 must be either l,l or d,d.

### Absolute configuration determination

Determination of the absolute configuration of x17299 was initially approached by its direct comparison to l,l-TMAP (**1c**) and *N,N,N*-trimethyl-d-alanyl-d-proline betaine (d,d-TMAP, **1b**). d,d-TMAP was chemically prepared following the same procedure for **1a** synthesis but starting with d-proline *t*-butyl ester (**11b**) instead. The intermediate d-alanyl-d-proline (**12b**) was characterized by LC-MS/MS (Figs. S24, S25). The reaction product **1b** was characterized by high resolution mass spectrometry with a protonated ion of *m/z* 229.1545 (−0.8 ppm off calculated value, Fig. S26). The MS^2^ (Fig. 2m; Table S6) and MS^3^ spectra of d,d-TMAP were identical to those of l,l-TMAP (Figs. S27, S28, S29). As expected, d,d-TMAP had the same chromatographic retention as l,l-TMAP and x17299 under achiral chromatographic conditions (Fig. 1d). It also co-eluted with l,l-TMAP and x17299 when co-injected (Figs. S30, S31).

**Fig. 3:**
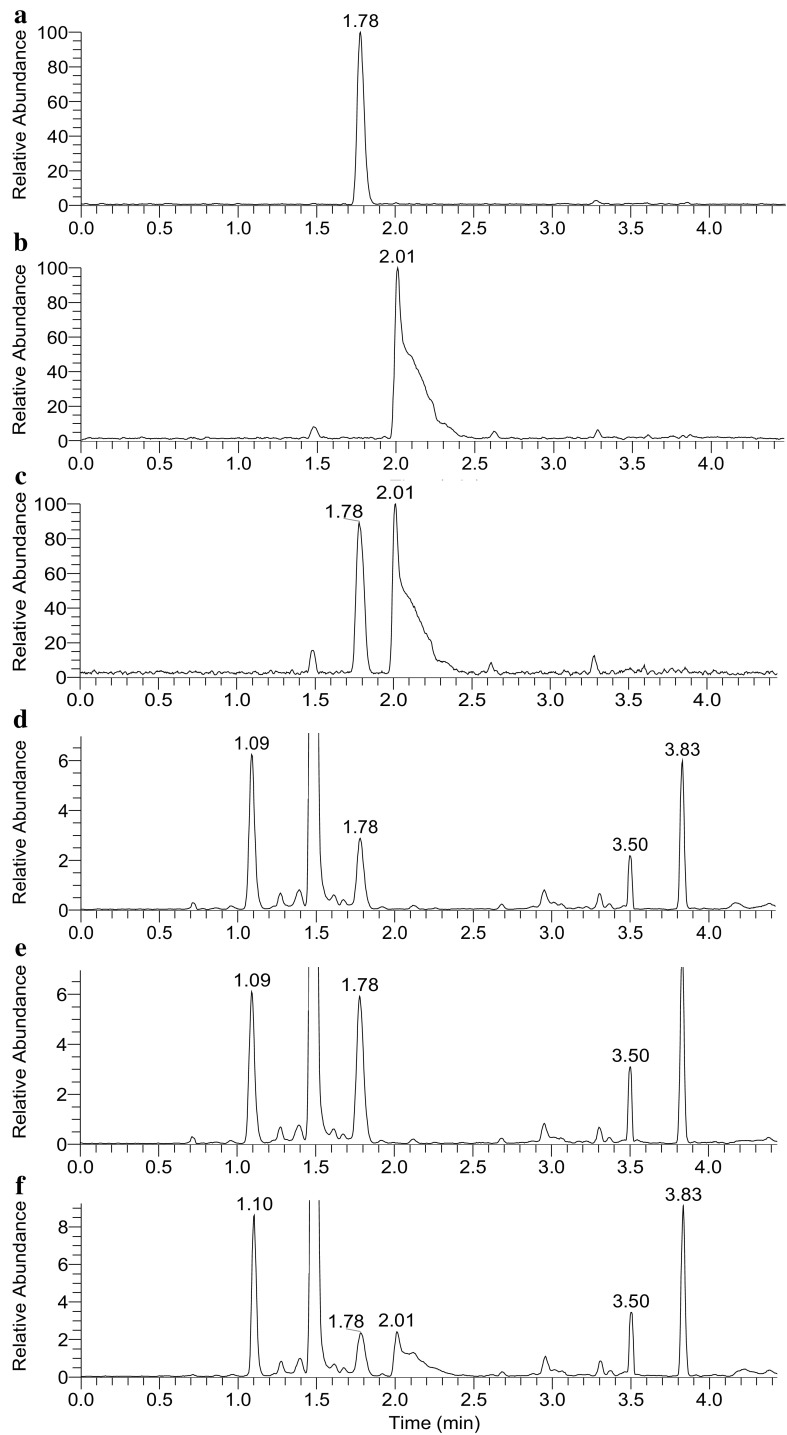
LC-MS/MS chromatograms of synthetic l,l-TMAP-l-Phe-OMe (1.78 min, **a**), d,d-TMAP-l-Phe-OMe (2.01 min, **b**), a mixture of synthetic l,l-TMAP-l-Phe-OMe and d,d-TMAP-l-Phe-OMe (**c**), x17299-l-Phe-OMe (1.78 min, **d**), a mixture of x17299-l-Phe-OMe and synthetic l,l-TMAP-l-Phe-OMe (**e**), and a mixture of x17299-l-Phe-OMe and synthetic d,d-TMAP-l-Phe-OMe (**f**)

Direct chromatographic resolution of l,l-TMAP and d,d-TMAP was tested unsuccessfully on a number of columns with chiral stationary phase, including CHIRALPAK^®^ AS-3R and CHIRALBIOTIC™ T. The enantiomers were then individually coupled to L-phenylalanine methyl ester (**14**) to give l,l-TMAP-l-Phe-OMe (**15c**) and d,d-TMAP-l-Phe-OMe (**15b**) as a pair of diastereomers (Scheme 2). Baseline separation of the diastereomers was achieved with a reversed phase chromatography method. l,l-TMAP-l-Phe-OMe (**15c**) eluted at 1.78 min with a nice peak shape (Fig. 3a) while d,d-TMAP-l-Phe-OMe (**15b**) eluted later at 2.01 min with a tailing peak shape (Fig. 3b). No epimerization products were detected in either reaction mixture, and the two derivatives well separated when co-injected (Fig. 3c). The expected protonated ion was observed at *m/z* 390.2389 (0.4 ppm off calculated value) and 390.2392 (1.1 ppm off calculated value) for the **15c** and **15b**, respectively (Fig. S32). The product ion spectra of the two compounds are very similar (Fig. S33; Tables S7, S8), so are the MS^3^ spectra of *m/z* 331, 271, 243, 303, 168, and 142 (Figs. S34, S35). Loss of trimethylamine from the alanyl moiety in the coupling product may generate the predominant *m/z* 331 ion, which then loses the ester group from the phenylalanine moiety to give *m/z* 271 ion (Scheme S1). These results support the identity of the coupling products.

When a concentrated plasma extract was treated with l-phenylalanine methyl ester (**14**) in the presence of EDC, a new peak was detected at 1.78 min (Fig. 3d). A protonated ion of *m/z* 390.2398 (2.7 ppm off calculated value) was detected for the coupling product of x17299-l-Phe-OMe (Fig. S36). The MS^2^ (Fig. S33; Table S9) and MS^3^ spectra of this coupling product are virtually superimposable on those of **15c** (Figs. S37, S38, S39). To demonstrate that x17299 was converted to x17299-l-Phe-OMe under the experimental conditions, l,l-TMAP-*d*_9_, which was synthesized with iodomethane-*d*_3_ and characterized (Fig. S40; Table S10), was spiked into to a plasma extract at a concentration comparable to that of x17299 (Fig. S41). After the spiked plasma extract was treated under the same coupling conditions, l,l-TMAP-*d*_9_-l-Phe-OMe was detected along with the co-eluting x17299-l-Phe-OMe (Figs. S41, S42; Table S11). The peak height ratio of these two reaction products is essentially the same as that of the two starting compounds in the spiked plasma extract (Fig. S41), indicating that x17299 was indeed converted to x17299-l-Phe-OMe under the experimental conditions. When **15c** and **15b** were individually spiked into the early reaction product mixture, co-elution of x17299-l-Phe-OMe and **15c** was observed at 1.78 min (Fig. 3e), while **15b** eluted much later at 2.01 min (Fig. 3f). These results clearly indicate that x17299-l-Phe-OMe is identical to **15c**, and thus x17299 itself has the l,l-configuration.

## Conclusion

The complete structure of x17299 was determined to be l,l-TMAP (**1c**) without any purification and NMR analysis. The gross structure of x17299 was elucidated by mechanistic interpretation of mass spectrometric data with the conducive consideration that this compound has a biological origin. Deuterium-exchanged MS experiments were shown to be a quick and valuable tool for the structure verification. Relative configuration of x17299 was determined by direct chromatographic comparison to a pair of synthetic diastereomers. Absolute configuration was assigned after derivatization of x17299 with a chiral auxiliary group followed by its chromatographic comparison to a pair of synthetic standards. It is noteworthy that a similar strategy is still required for stereochemistry determination even if NMR analysis is used for the structure elucidation of x17299. Our results demonstrate that de novo interpretation of mass spectrometric data can be a convenient and valuable, albeit still challenging, approach for structure elucidation of certain unknown metabolites in metabolomic studies. This is particularly suited for some polar metabolites with low molecular weight and trace concentration, where purification for NMR analysis is difficult while chemical synthesis for structure confirmation is often straightforward.

## Electronic supplementary material

Below is the link to the electronic supplementary material.

Supplementary material 1 (PDF 2624 KB)
