# Supplementary material for: Structure elucidation of metabolite x17299 by interpretation of mass spectrometric data
Source: Metabolomics. 2017 Jun 24;13(8):92. doi: 10.1007/s11306-017-1231-x (PMC5486616; doi:10.1007/s11306-017-1231-x)
Supplement: Supplementary file 1 — Supplementary material 1 (PDF 2624 KB) [file 11306_2017_1231_MOESM1_ESM.pdf]

Supplementary Material

Structure Elucidation of Metabolite x17299 by Interpretation of Mass Spectrometric Data

Qibo Zhang,* Lisa A. Ford, Anne M. Evans, Douglas R. Toal

Metabolon, Inc., 617 Davis Drive, Suite 400, Morrisville, NC 27560, USA

Corresponding author:

*Tel: +1 (919) 595-2287. E-mail: qzhang@metabolon.com.

Supporting Information Table of Contents

Figure S1. LC/MS chromatogram and spectrum of x17299 (1.44 min) in a plasma extract with expansion (top)....S-4

Figure S2. MS^4 spectrum of m/z 229/142/114 of x17299. ....S-4

Figure S3. LC/MS chromatogram and spectrum of deuterated x17299 (1.57 min) with expansion (top)....S-5

Figure S4. MS^3 spectrum of m/z 230/143 of deuterated x17299 with expansion (top)....S-6

Figure S5. LC/MS chromatogram and spectrum of synthetic L,L-TMAP (1.44 min). S-6

Figure S6. LC-MS/MS chromatograms of x17299 (1.44 min) in a plasma extract (top), synthetic L,L-TMAP (1.44 min, middle), and their co-injection (bottom). S-7

Figure S7. MS^2 spectra of x17299 (left) and synthetic L,L-TMAP (right) with expansions (top and middle). S-8

Figure S8. MS^3 spectra of m/z 229/142 of x17299 (left) and synthetic L,L-TMAP (right) with expansion (top). S-9

Figure S9. MS^3 spectra of m/z 229/170 of x17299 (left) and synthetic L,L-TMAP (right) with expansion (top). S-9

Figure S10. MS^4 spectra of m/z 229/142/114 of synthetic L,L-TMAP (bottom) and x17299 (top) for comparison. S-10

Figure S11. MS spectrum of deuterated synthetic L,L-TMAP with expansion (top). S-10

Figure S12. MS^2 spectra of deuterated x17299 (left) and synthetic L,L-TMAP (right) with expansions (top and middle). S-11

Figure S13. MS^3 spectra of m/z 230/143 of deuterated x17299 (left) and synthetic L,L-TMAP (right) with expansion (top). S-11

Figure S14. LC/MS chromatogram and spectrum of synthetic D-alanyl-L-proline (2.24 min). S-12

Figure S15. LC/MS chromatogram and spectrum of L-alanyl-L-proline (1.50 min) from TCI for comparison. S-12

Figure S16. MS^2 spectrum of synthetic D-alanyl-L-proline with expansion (top). S-13

Figure S17. MS^2 spectrum of L-alanyl-L-proline from TCI with expansion (top) for comparison. S-13

Figure S18. LC-MS/MS chromatograms of D-alanyl-L-proline (top, 2.25 min, synthesized), L-alanyl-L-proline (middle, 1.50 min, from TCI), and a mixture of the two (bottom). S-14

Figure S19. LC/MS chromatogram and spectrum of synthetic D,L-TMAP (2.37 min). S-15

Figure S20. MS^2 spectra of synthetic D,L-TMAP (left) and L,L-TMAP (right) with expansions (top and middle) for comparison. S-15

Figure S21. MS^3 spectra of m/z 229/142 of synthetic D,L-TMAP (left) and L,L-TMAP (right) with expansion (top) for comparison. S-16

Figure S22. MS^3 spectra of m/z 229/170 of synthetic D,L-TMAP (left) and L,L-TMAP (right) with expansion (top) for comparison. S-16

Figure S23. LC-MS/MS chromatograms of x17299 (1.42 min) in a plasma extract (top), synthetic D,L-TMAP (2.36 min, middle), and their co-injection (bottom). S-17

Figure S24. LC/MS chromatogram and spectrum of synthetic D-alanyl-D-proline (1.50 min). S-18

Figure S25. MS^2 spectrum of synthetic D-alanyl-D-proline with expansion (top). S-18

Figure S26. LC/MS chromatogram and spectrum of synthetic D,D-TMAP (1.45 min). S-19

Figure S27. MS^2 spectra of synthetic D,D-TMAP (left) and L,L-TMAP (right) with expansions (top and middle) for comparison. S-20

Figure S28. MS^3 spectra of m/z 229/142 of synthetic D,D-TMAP (left) and L,L-TMAP (right) with expansion (top) for comparison. S-20

Figure S29. MS^3 spectra of m/z 229/170 of synthetic D,D-TMAP (left) and L,L-TMAP (right) with expansion (top) for comparison. S-21

Figure S30. LC-MS/MS chromatograms of synthetic D,D-TMAP (1.41 min, top), L,L-TMAP (1.41 min, middle), and their co-injection (bottom). S-21

Figure S31. LC-MS/MS chromatograms of synthetic D,D-TMAP (1.41 min, top), x17299 (1.41 min, middle) in a plasma extract, and their co-injection (bottom). S-22

Figure S32. LC/MS chromatograms and mass spectra of L,L-TMAP-L-Phe-OMe (1.78 min, left) and D,D-TMAP-L-Phe-OMe (2.01 min, right) with expansions (top). S-23

Figure S33. MS^2 spectra of L,L-TMAP-L-Phe-OMe (middle), D,D-TMAP-L-Phe-OMe (bottom), and x17299-L-Phe-OMe (top). S-24

Figure S34. MS^3 spectra of m/z 390/331 (top), m/z 390/271 (middle), and m/z 390/243 (bottom) of L,L-TMAP-L-Phe-OMe (left) and D,D-TMAP-L-Phe-OMe (right). S-25

Figure S35. MS^3 spectra of m/z 390/303 (top), m/z 390/168 (middle), and m/z 390/142 (bottom) of L,L-TMAP-L-Phe-OMe (left) and D,D-TMAP-L-Phe-OMe (right). S-26

Figure S36. LC/MS chromatograms and mass spectrum of x17299-L-Phe-OMe (1.77 min) with expansion (top). S-27

Figure S37. MS^3 spectra of m/z 390/331 of x17299-L-Phe-OMe (top), L,L-TMAP-L-Phe-OMe (middle), and D,D-TMAP-L-Phe-OMe (bottom). S-28

Figure S38. MS^3 spectra of m/z 390/271 of x17299-L-Phe-OMe (top), L,L-TMAP-L-Phe-OMe (middle), and D,D-TMAP-L-Phe-OMe (bottom). S-29

Figure S39. MS^3 spectra of m/z 390/243 of x17299-L-Phe-OMe (top), L,L-TMAP-L-Phe-OMe (middle), and D,D-TMAP-L-Phe-OMe (bottom). S-30

Figure S40. LC/MS chromatogram and MS spectrum (left), and MS^2 spectrum (right) of synthetic L,L-TMAP-d_9 (1.43 min) with expansions (middle and top). S-31

Figure S41. LC/MS chromatograms of x17299 (1.45 min, left top) and L,L-TMAP-d_9 (1.44 min, left bottom) in a spiked plasma extract, and x17299-L-Phe-OMe (1.71 min, right top) and L,L-TMAP-d_9-L-Phe-OMe (1.71 min, right bottom) in a coupling reaction mixture of the spiked plasma extract. S-32

Figure S42. MS (top) and MS^2 (bottom) spectra of L,L-TMAP-d_9-L-Phe-OMe. S-32

Scheme S1. Proposed fragmentation pathways of TMAP-L-Phe-OMe. S-33

Table S1. Spectrum list of MS^2 of x17299. .... S-34

Table S2. Spectrum list of MS^2 of deuterated x17299. .... S-34

Table S3. Spectrum list of MS^2 of synthetic L,L-TMAP. S-35

Table S4. Spectrum list of MS^2 of deuterated synthetic L,L-TMAP. S-35

Table S5. Spectrum list of MS^2 of synthetic D,L-TMAP. S-36

Table S6. Spectrum list of MS^2 of synthetic D,D-TMAP. S-36

Table S7. Spectrum list of MS^2 of L,L-TMAP-L-Phe-OMe. S-37

Table S8. Spectrum list of MS^2 of D,D-TMAP-L-Phe-OMe. S-37

Table S9. Spectrum list of MS^2 of x17299-L-Phe-OMe. S-38

Table S10. Spectrum list of MS^2 of synthetic L,L-TMAP-d_9. .... S-38

Table S11. Spectrum list of MS^2 of L,L-TMAP-d_9-L-Phe-OMe. S-39

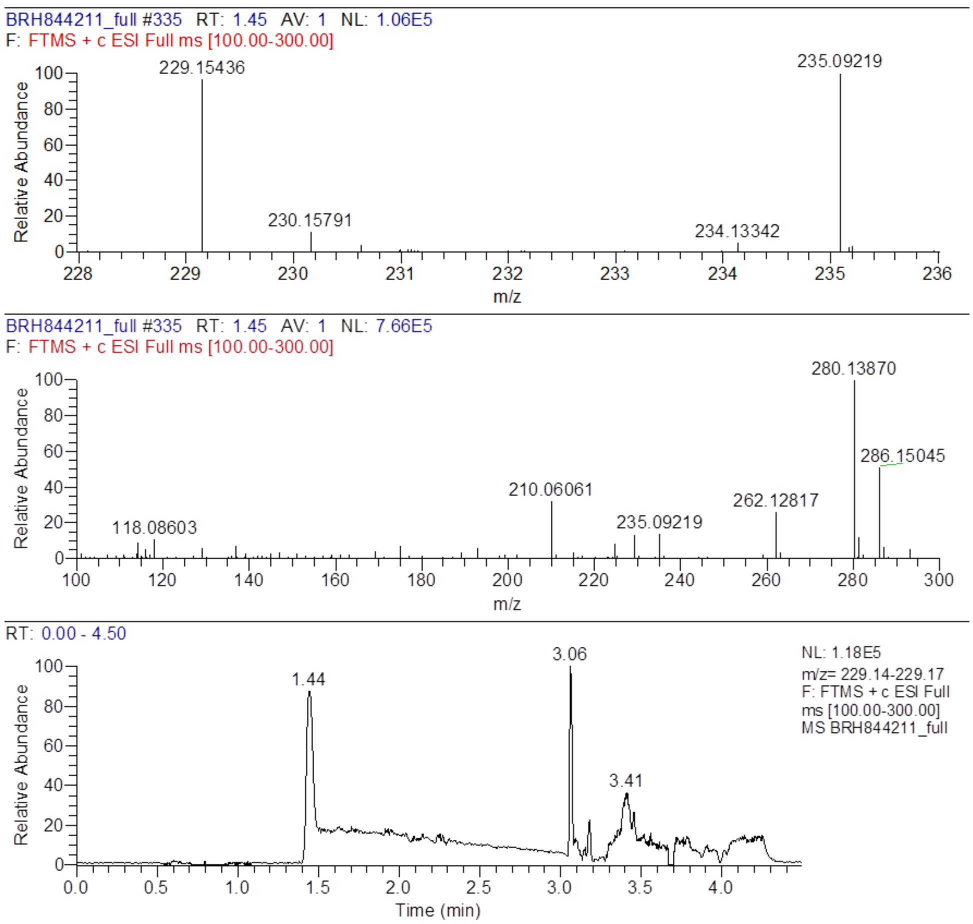

Figure S1. LC/MS chromatogram and spectrum of x17299 (1.44 min) in a plasma extract with expansion (top).

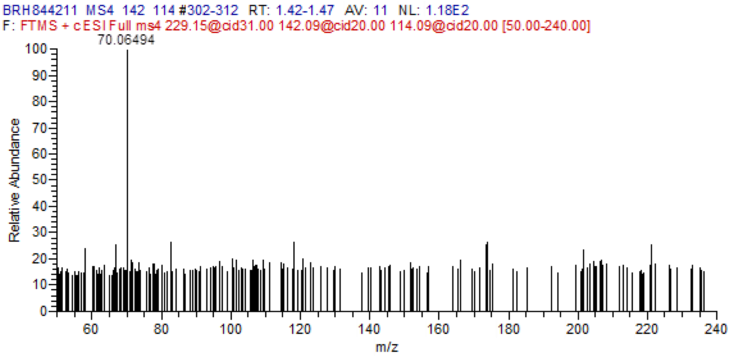

Figure S2. MS^4 spectrum of *m/z* 229/142/114 of x17299.

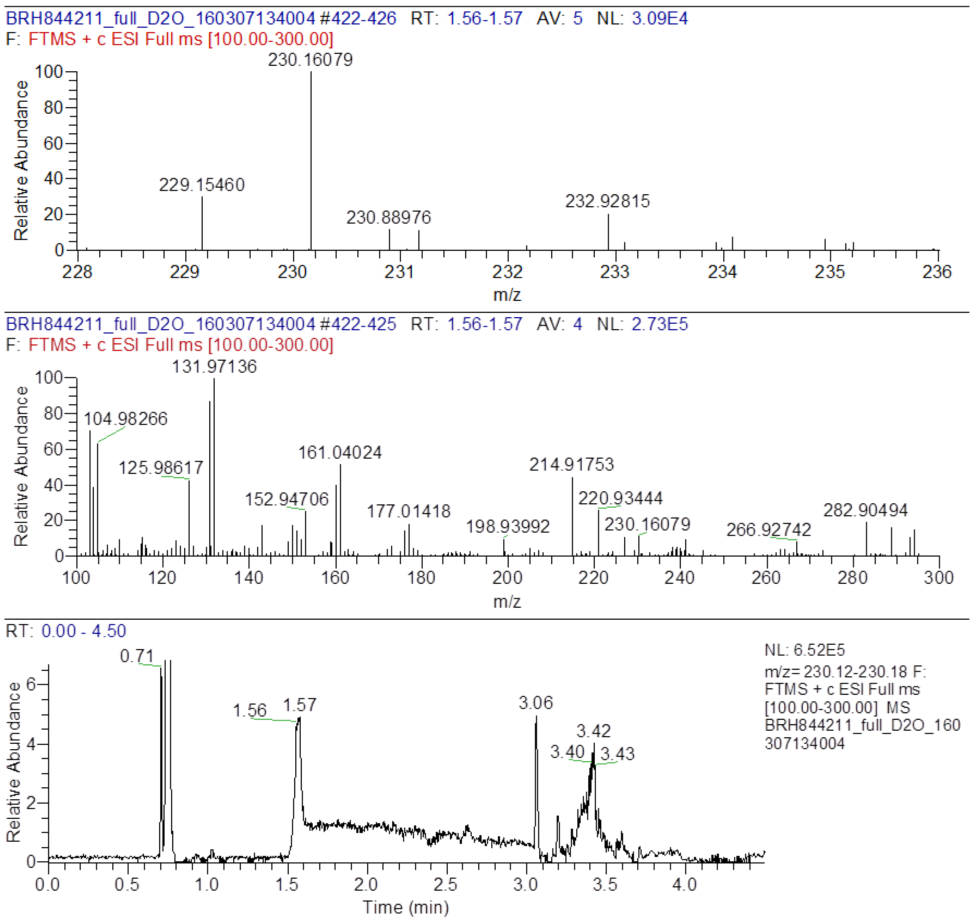

Figure S3. LC/MS chromatogram and spectrum of deuterated x17299 (1.57 min) with expansion (top).

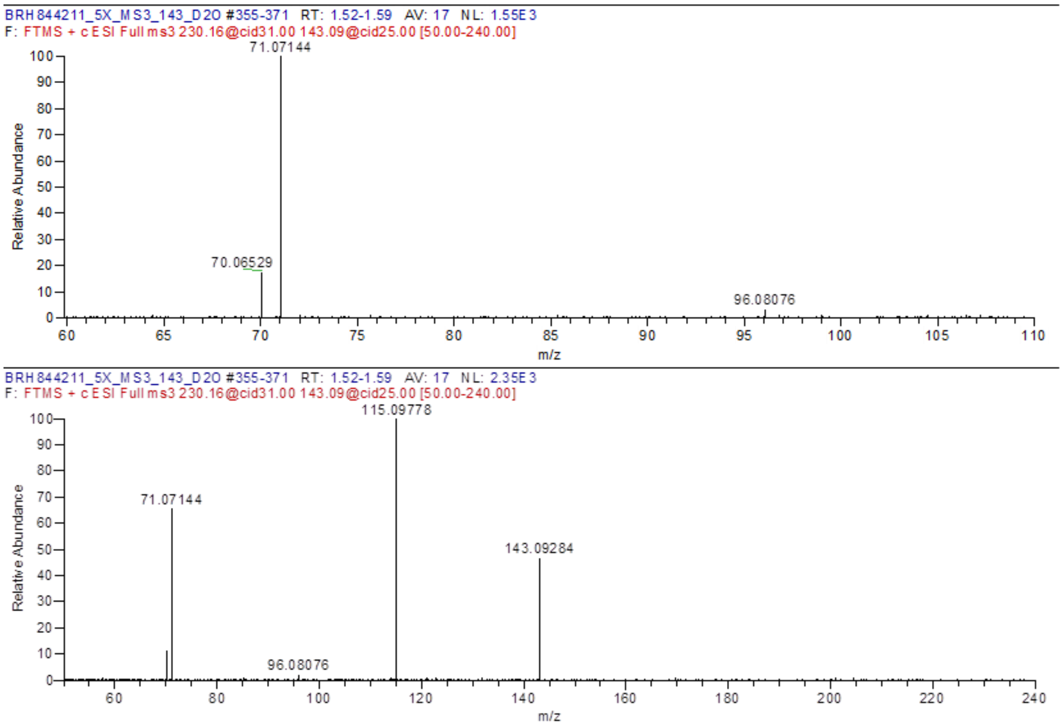

Figure S4. MS^3 spectrum of *m/z* 230/143 of deuterated x17299 with expansion (top).

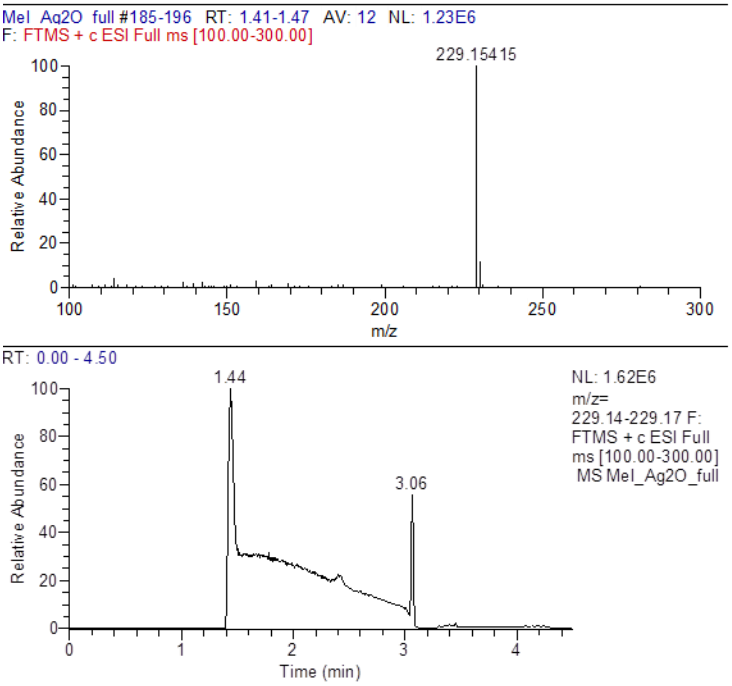

Figure S5. LC/MS chromatogram and spectrum of synthetic L,L-TMAP (1.44 min).

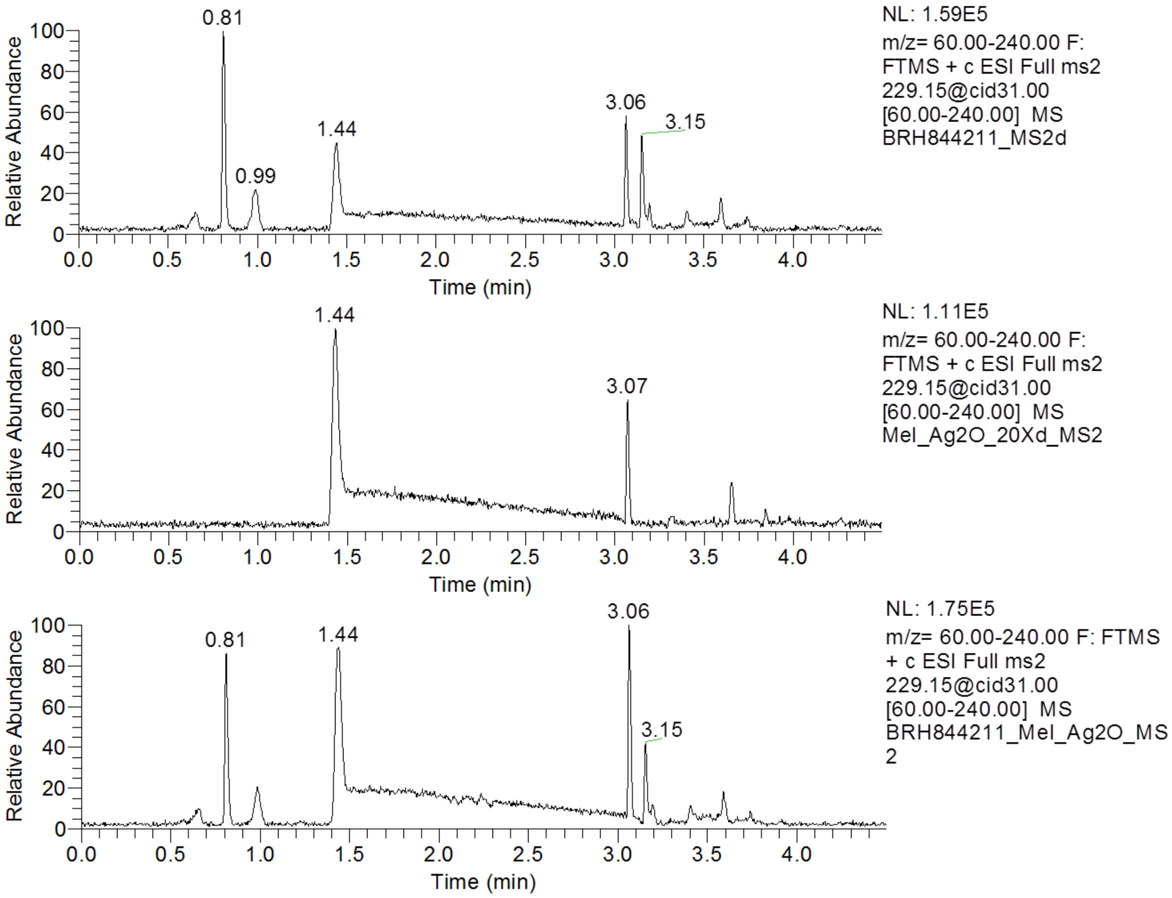

Figure S6. LC-MS/MS chromatograms of x17299 (1.44 min) in a plasma extract (top), synthetic L,L-TMAP (1.44 min, middle), and their co-injection (bottom).

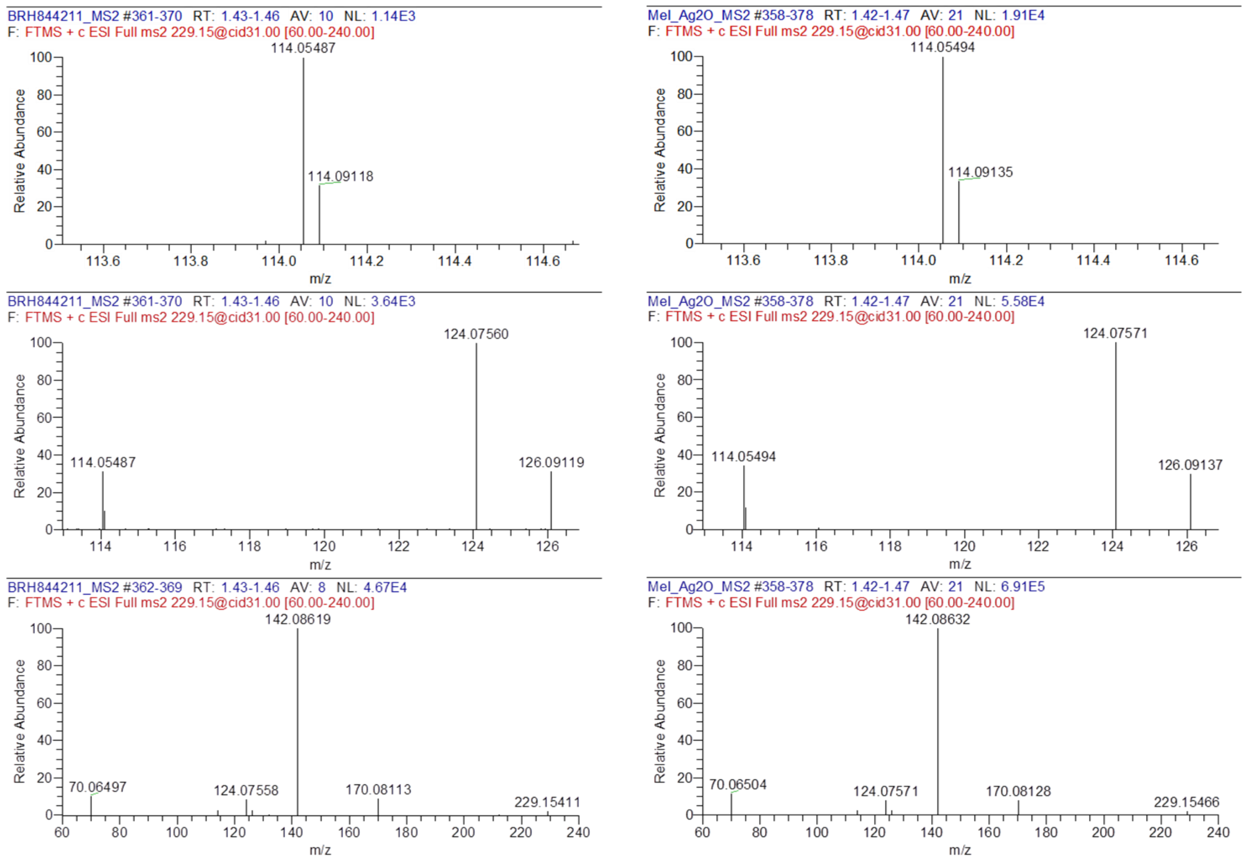

Figure S7. MS^2 spectra of x17299 (left) and synthetic L,L-TMAP (right) with expansions (top and middle).

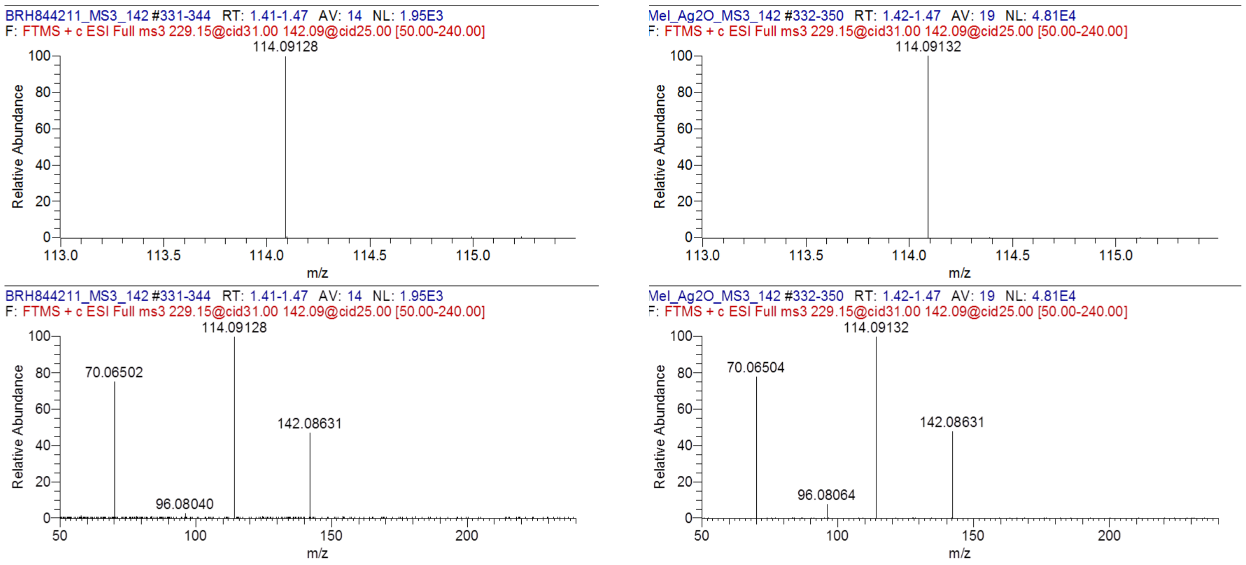

Figure S8. MS^3 spectra of *m/z* 229/142 of x17299 (left) and synthetic L,L-TMAP (right) with expansion (top).

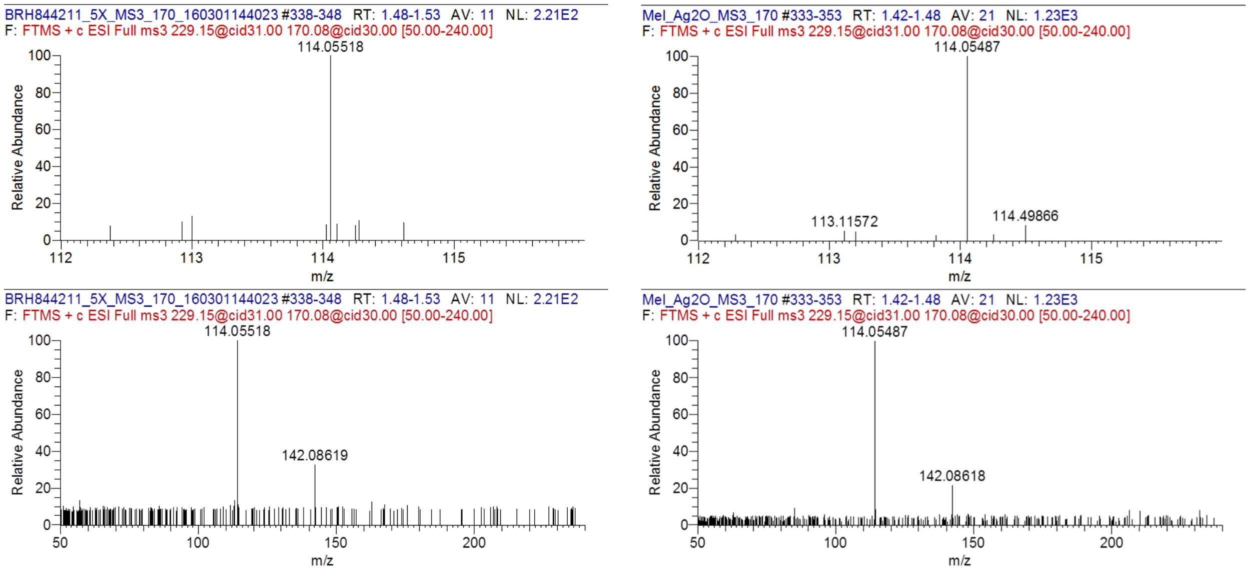

Figure S9. MS^3 spectra of *m/z* 229/170 of x17299 (left) and synthetic L,L-TMAP (right) with expansion (top).

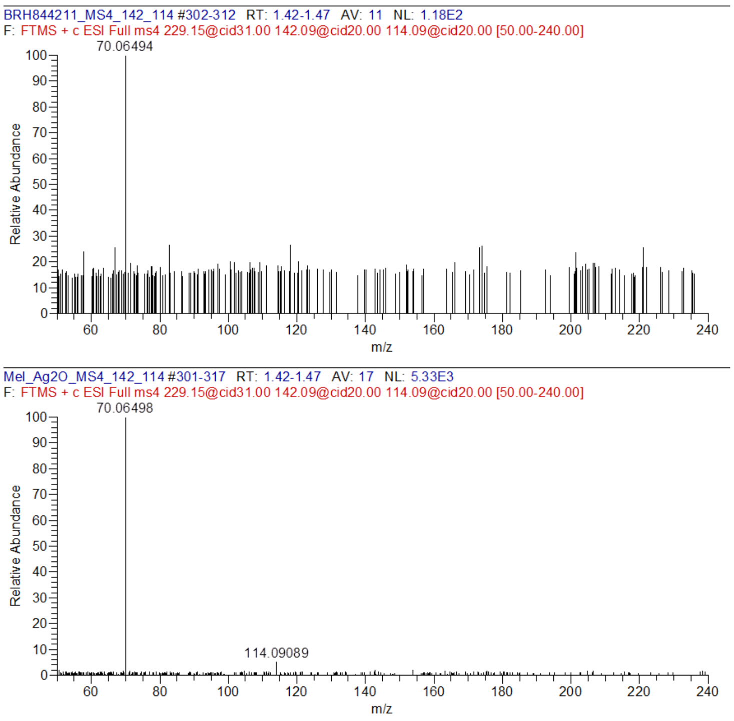

Figure S10. MS^4 spectra of *m/z* 229/142/114 of synthetic L,L-TMAP (bottom) and x17299 (top) for comparison.

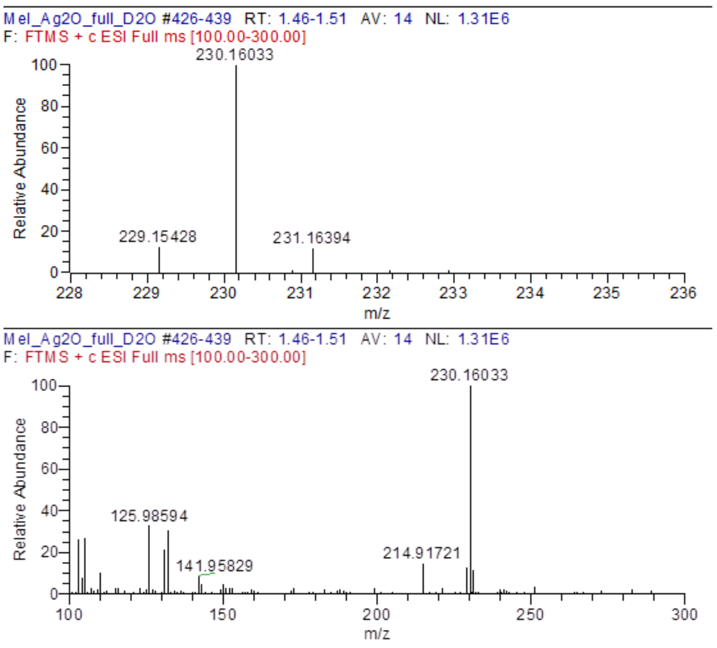

Figure S11. MS spectrum of deuterated synthetic L,L-TMAP with expansion (top).

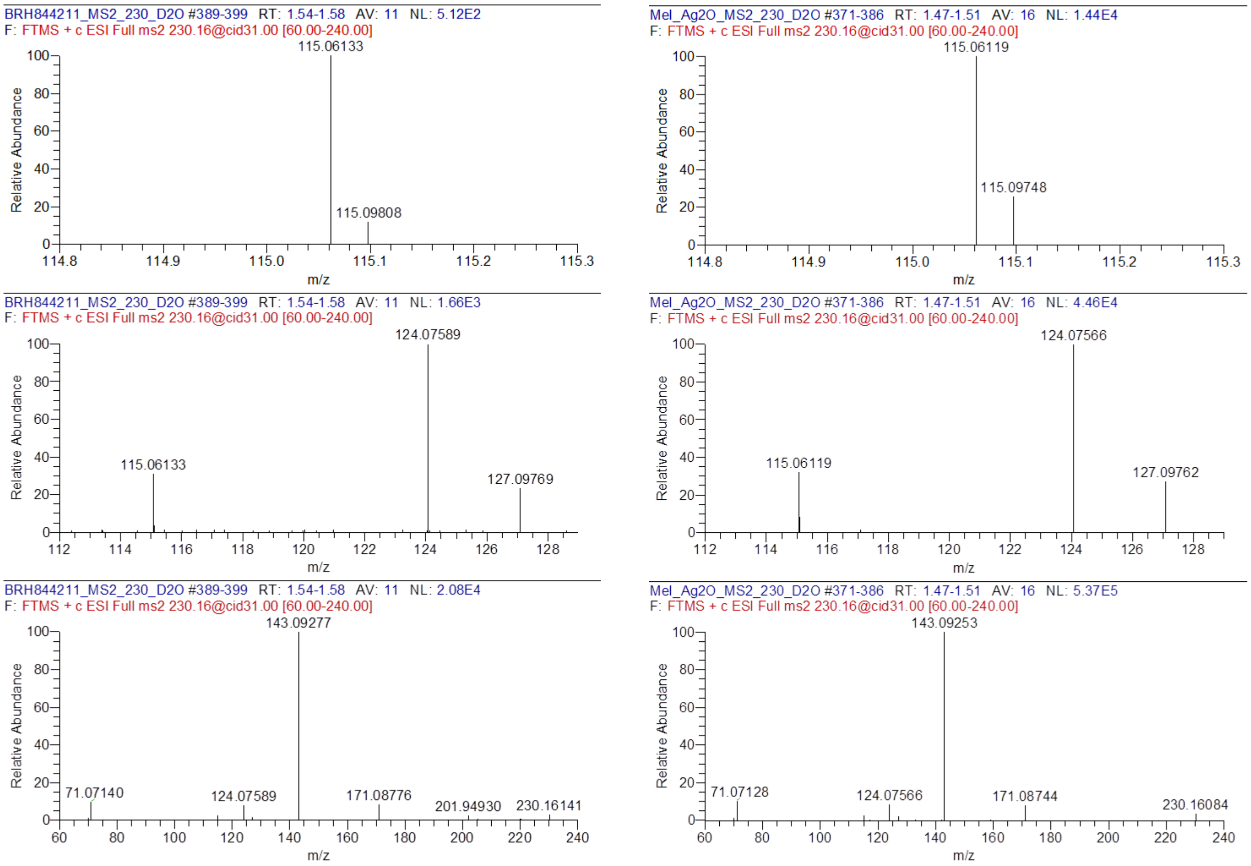

Figure S12. MS^2 spectra of deuterated x17299 (left) and synthetic L,L-TMAP (right) with expansions (top and middle).

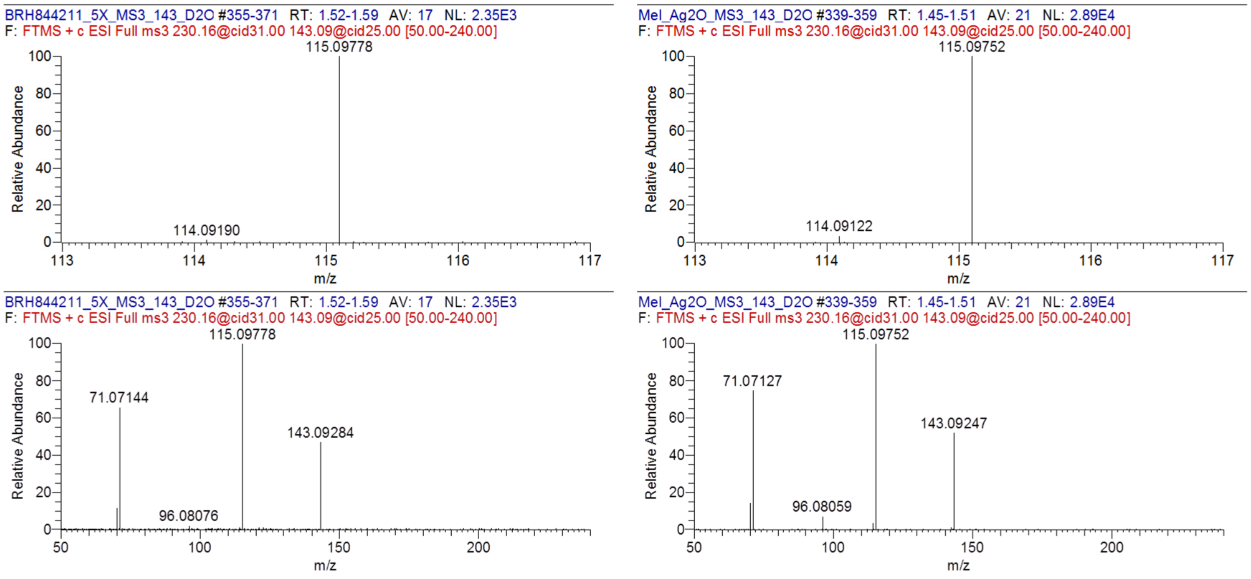

Figure S13. MS^3 spectra of *m/z* 230/143 of deuterated x17299 (left) and synthetic L,L-TMAP (right) with expansion (top).

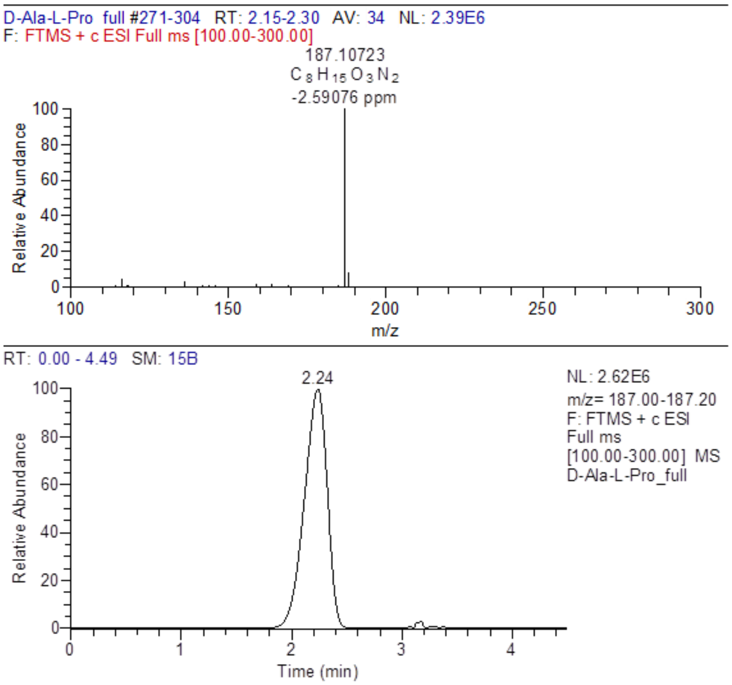

Figure S14. LC/MS chromatogram and spectrum of synthetic D-alanyl-L-proline (2.24 min).

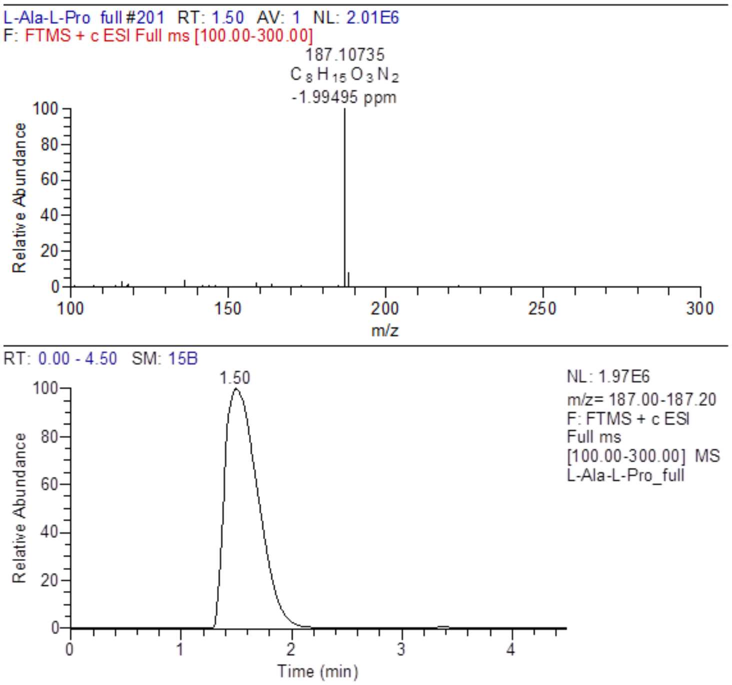

Figure S15. LC/MS chromatogram and spectrum of L-alanyl-L-proline (1.50 min) from TCI for comparison.

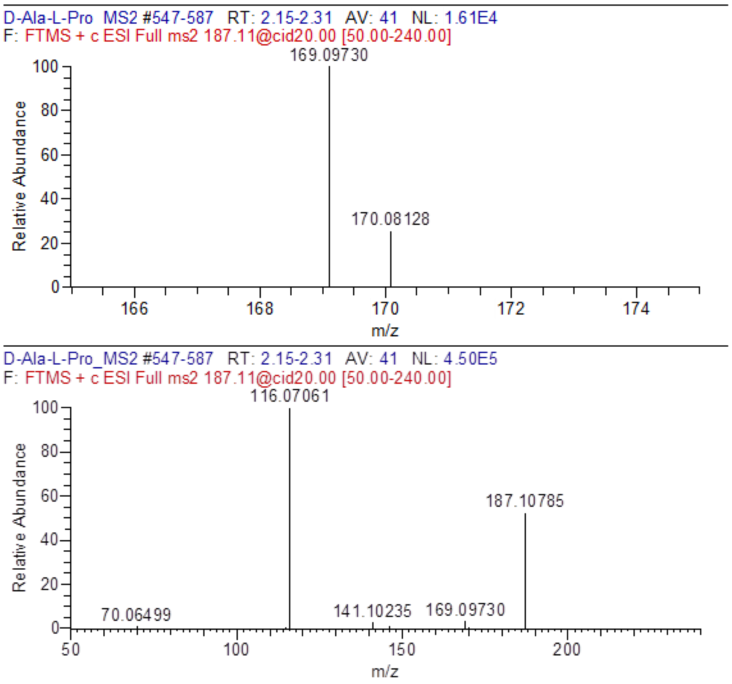

Figure S16. MS^2 spectrum of synthetic D-alanyl-L-proline with expansion (top).

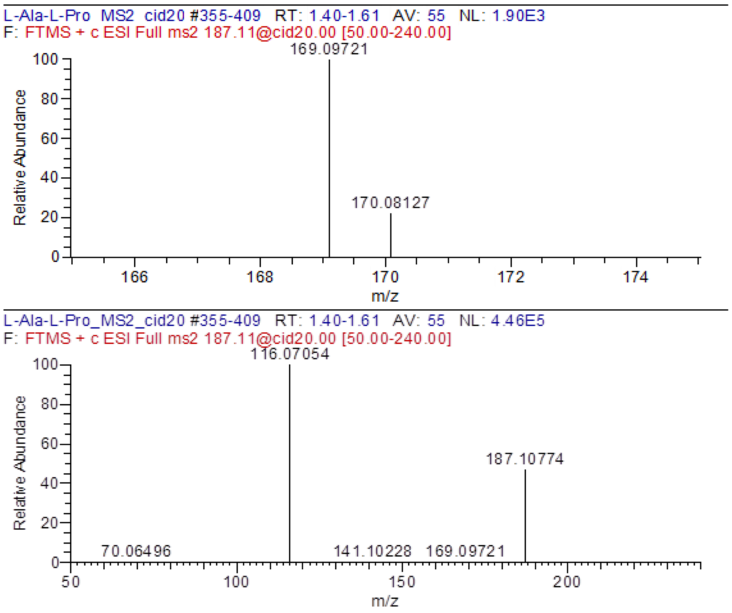

Figure S17. MS^2 spectrum of L-alanyl-L-proline from TCI with expansion (top) for comparison.

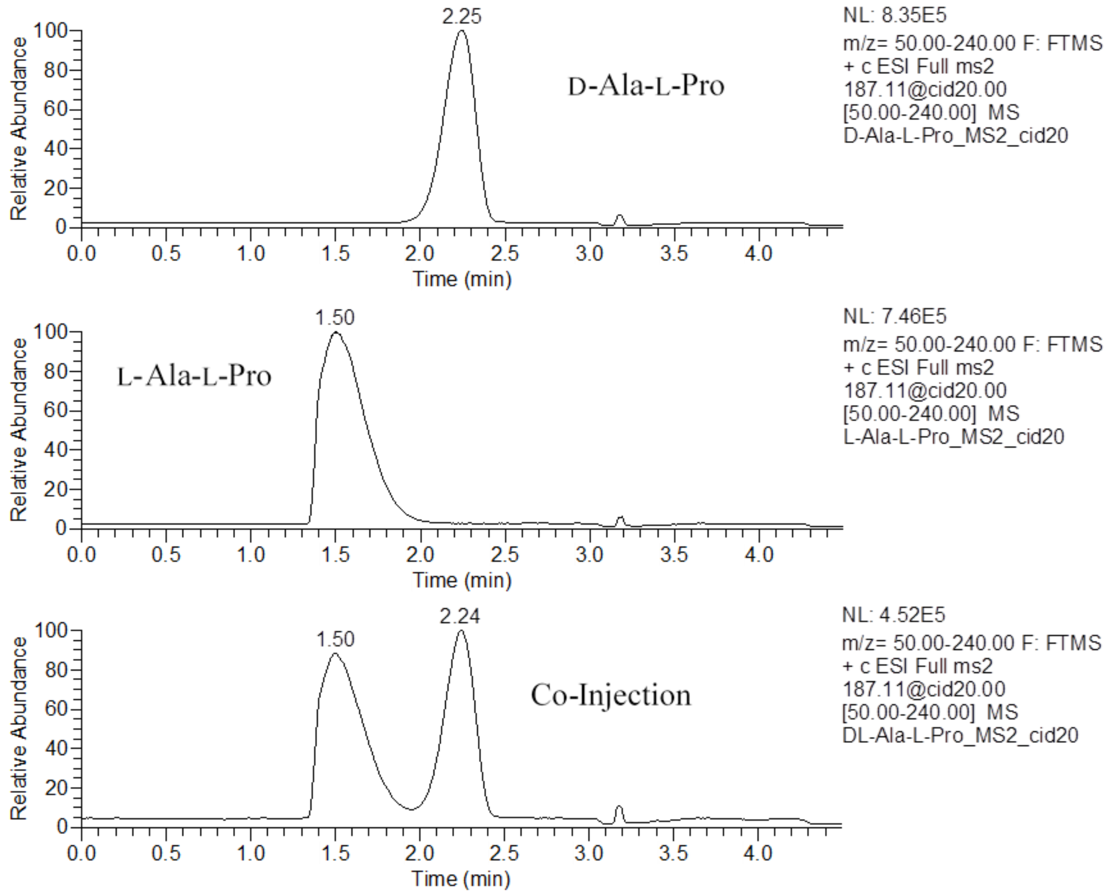

Figure S18. LC-MS/MS chromatograms of D-alanyl-L-proline (top, 2.25 min, synthesized), L-alanyl-L-proline (middle, 1.50 min, from TCI), and a mixture of the two (bottom).

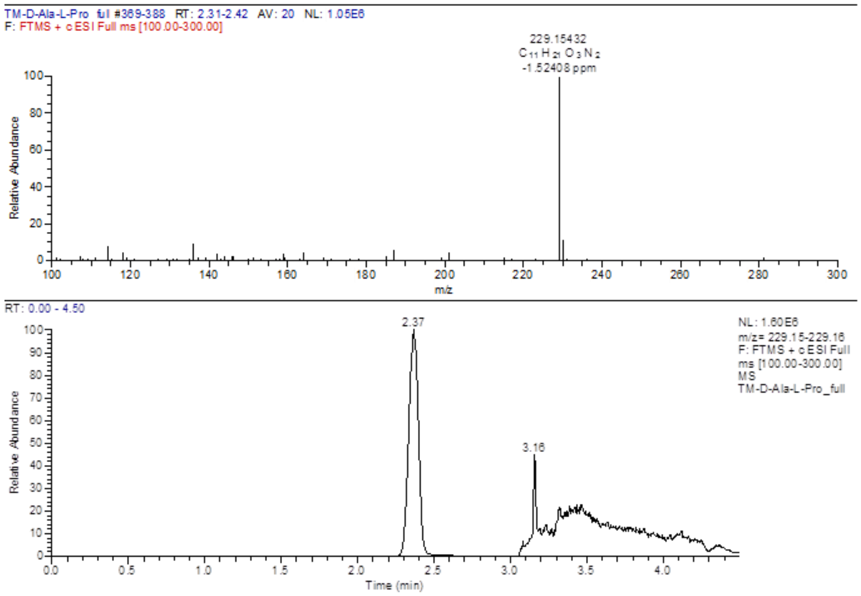

Figure S19. LC/MS chromatogram and spectrum of synthetic D,L-TMAP (2.37 min).

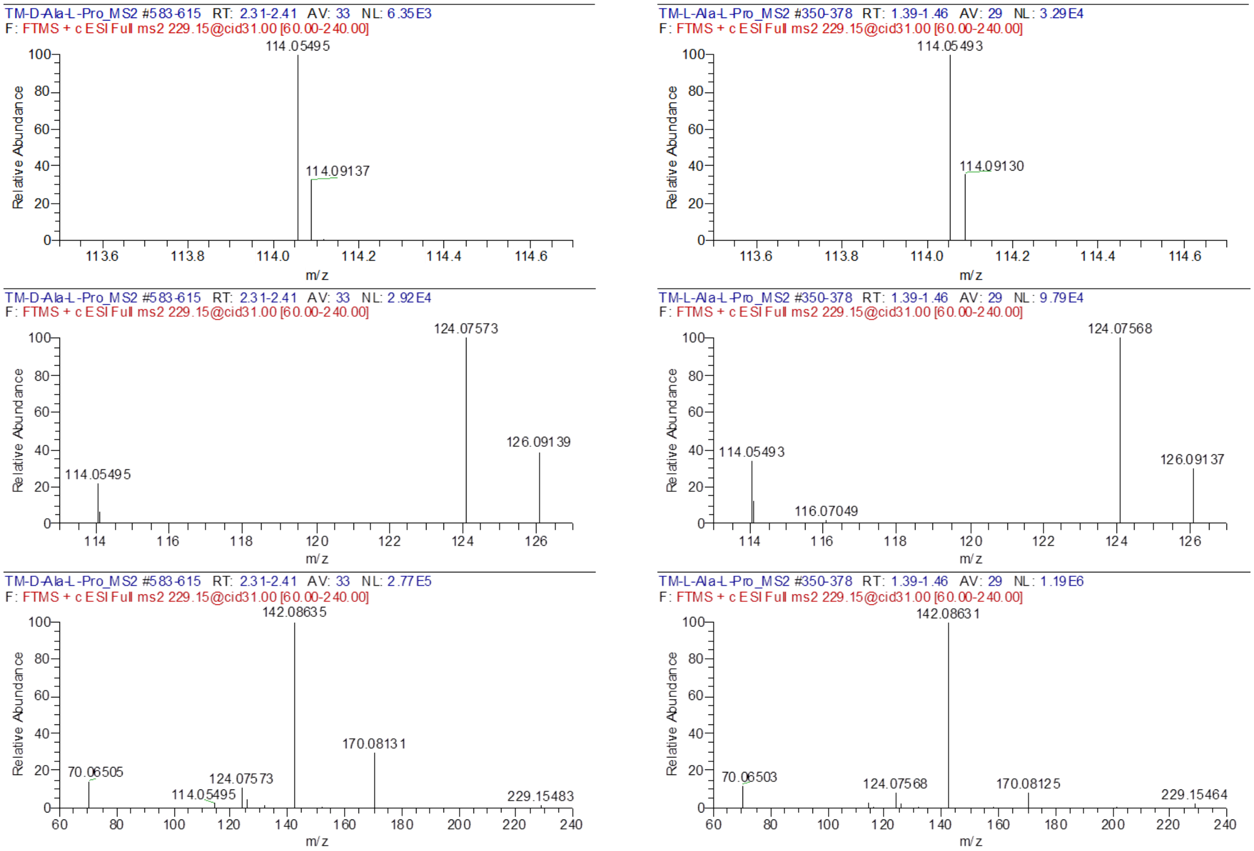

Figure S20. MS^2 spectra of synthetic D,L-TMAP (left) and L,L-TMAP (right) with expansions (top and middle) for comparison.

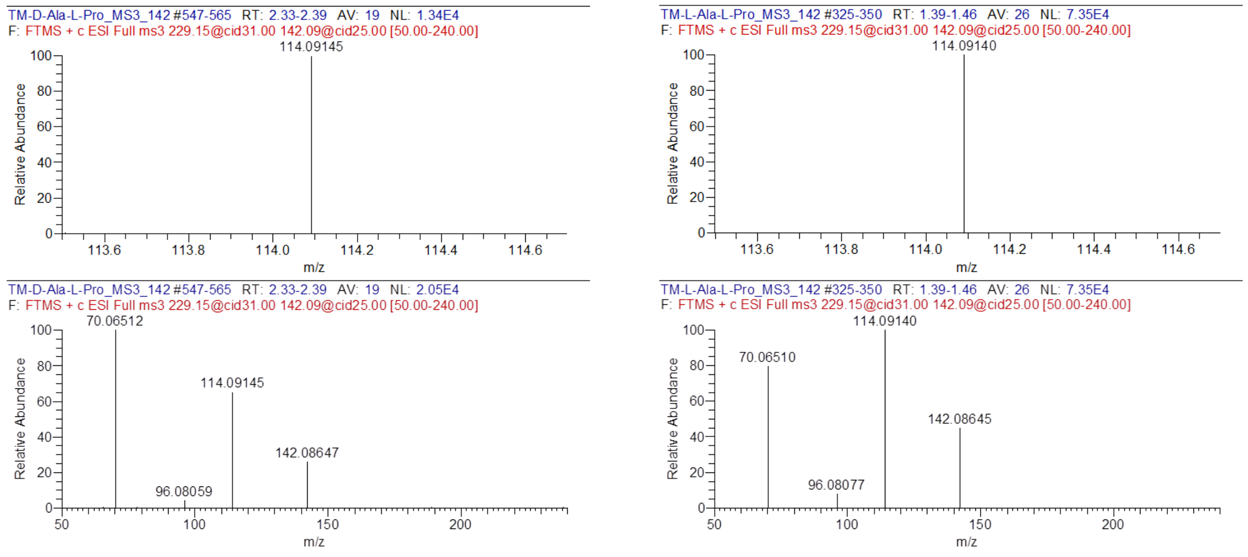

Figure S21. MS^3 spectra of *m/z* 229/142 of synthetic D,L-TMAP (left) and L,L-TMAP (right) with expansion (top) for comparison.

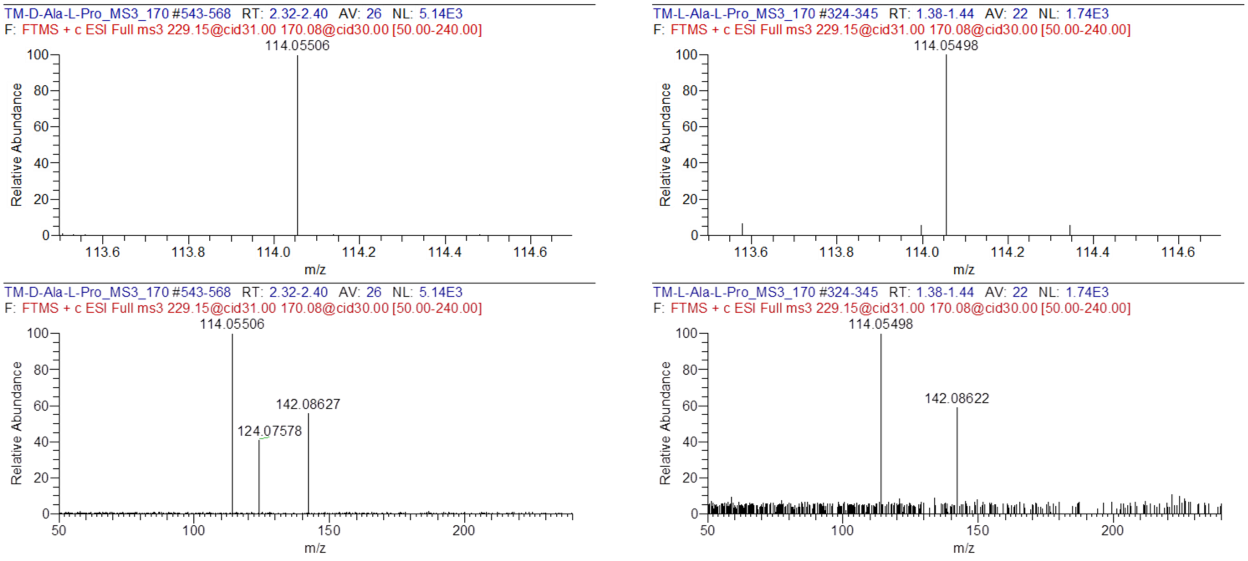

Figure S22. MS^3 spectra of *m/z* 229/170 of synthetic D,L-TMAP (left) and L,L-TMAP (right) with expansion (top) for comparison.

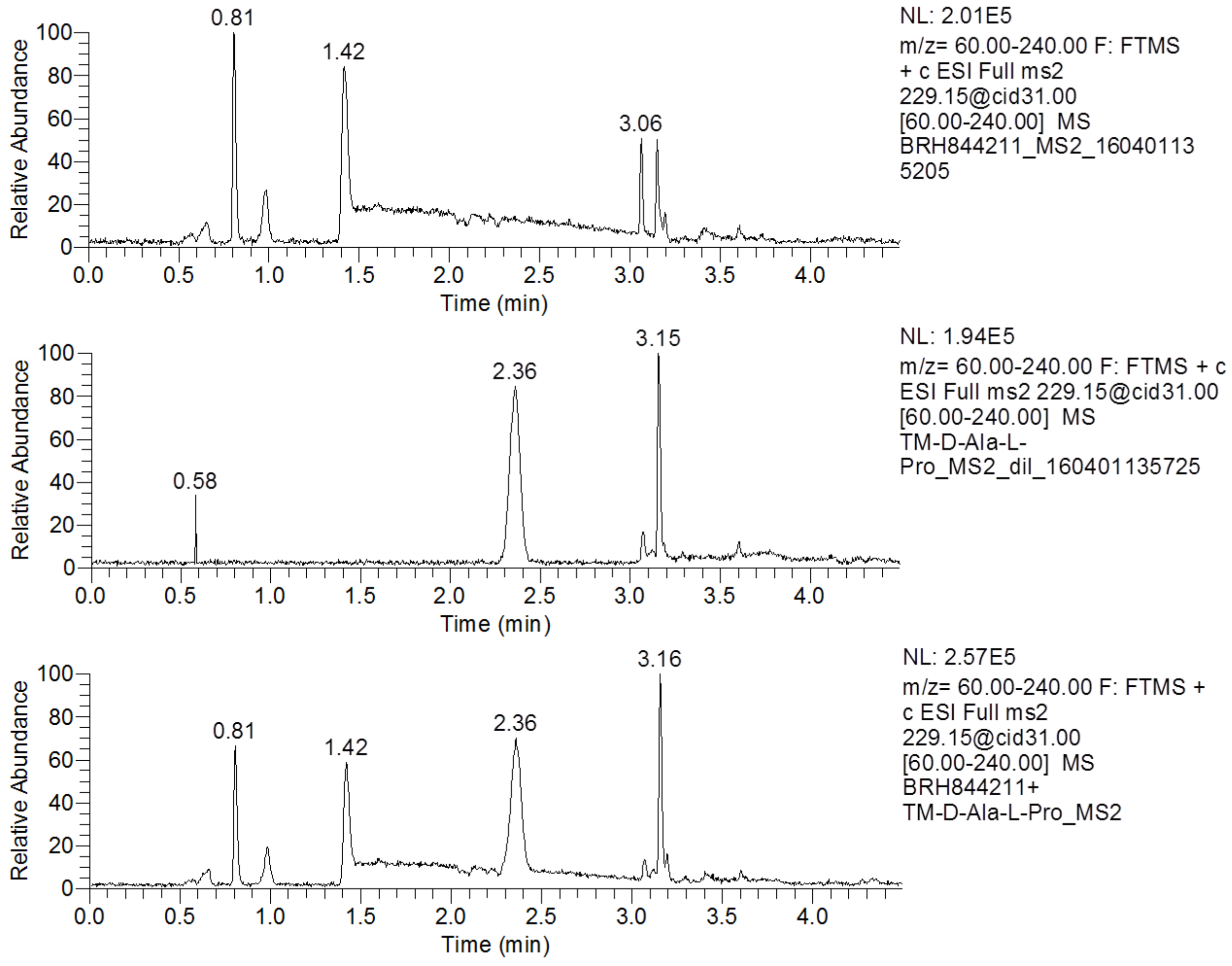

Figure S23. LC-MS/MS chromatograms of x17299 (1.42 min) in a plasma extract (top), synthetic D,L-TMAP (2.36 min, middle), and their co-injection (bottom).

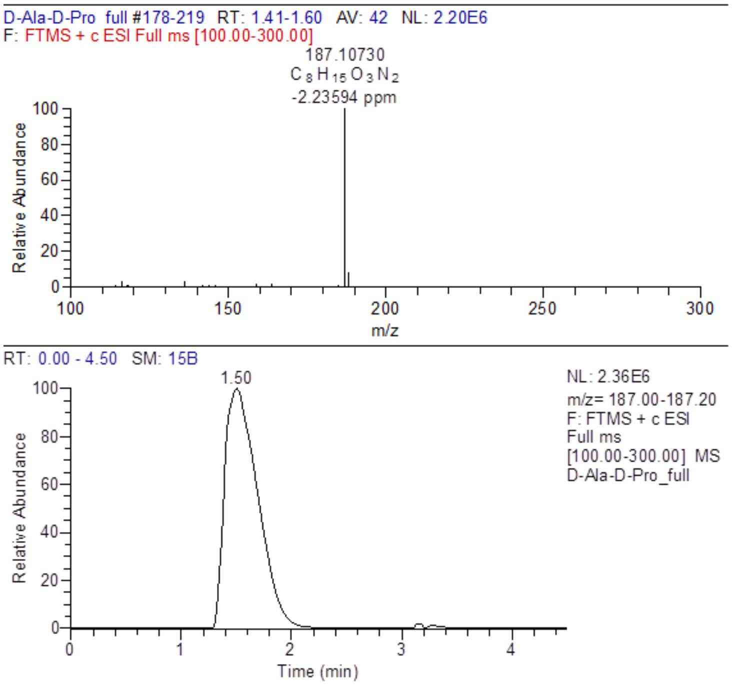

Figure S24. LC/MS chromatogram and spectrum of synthetic D-alanyl-D-proline (1.50 min).

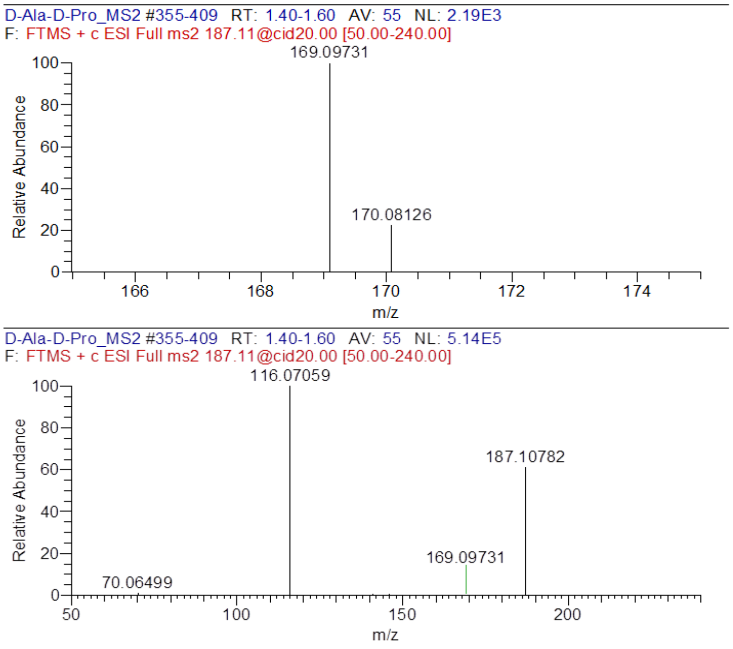

Figure S25. MS^2 spectrum of synthetic D-alanyl-D-proline with expansion (top).

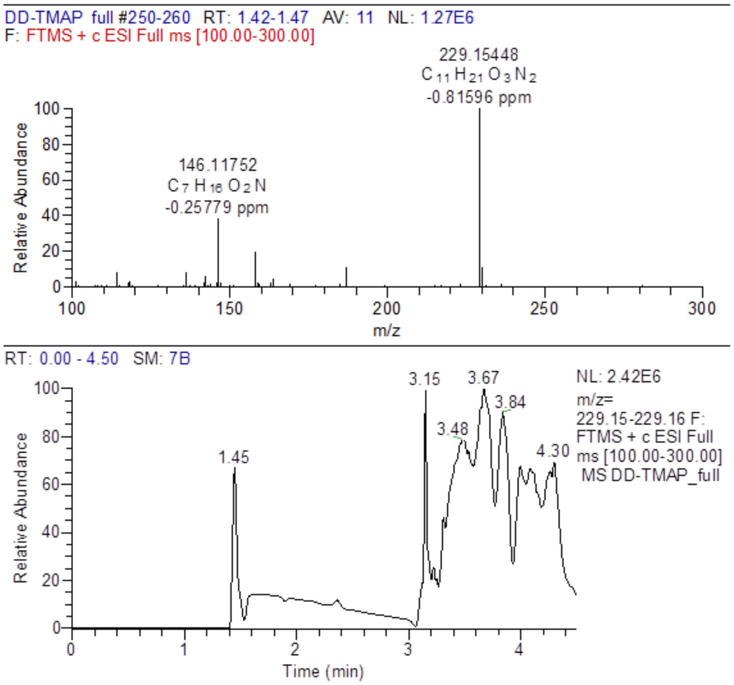

Figure S26. LC/MS chromatogram and spectrum of synthetic D,D-TMAP (1.45 min).

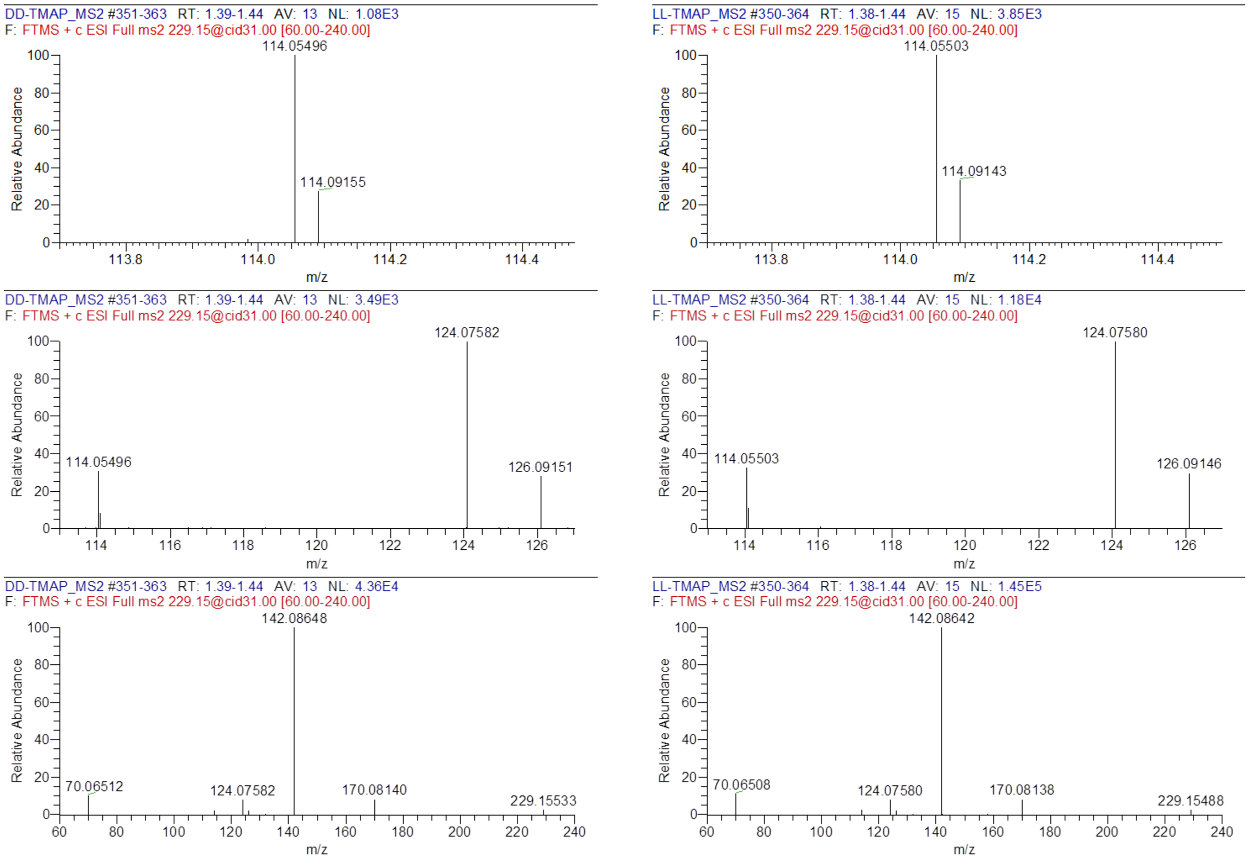

Figure S27. MS^2 spectra of synthetic D,D-TMAP (left) and L,L-TMAP (right) with expansions (top and middle) for comparison.

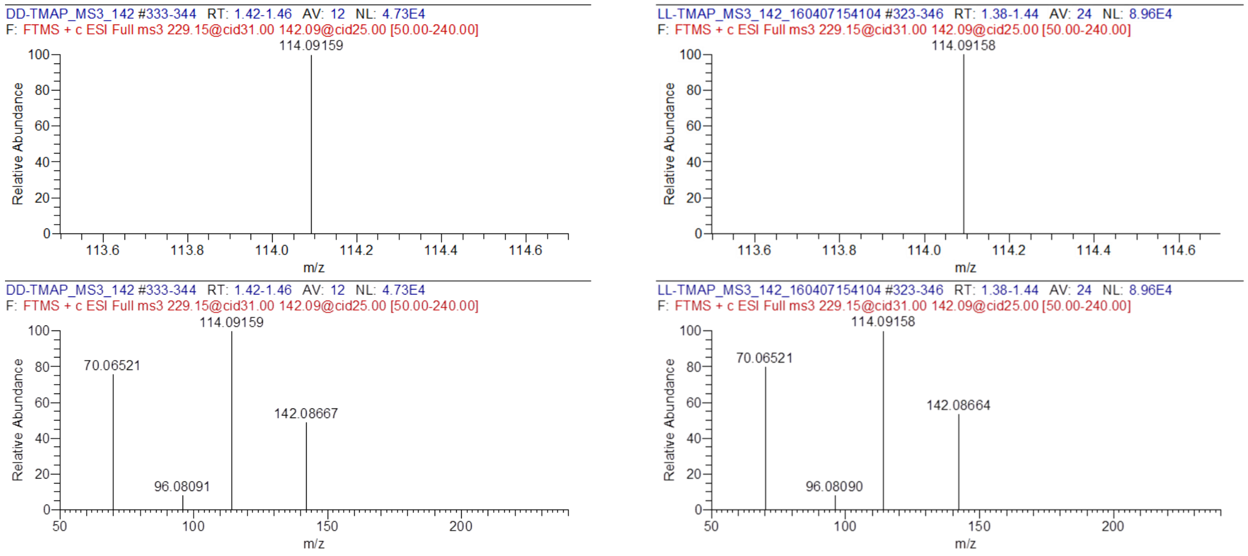

Figure S28. MS^3 spectra of *m/z* 229/142 of synthetic D,D-TMAP (left) and L,L-TMAP (right) with expansion (top) for comparison.

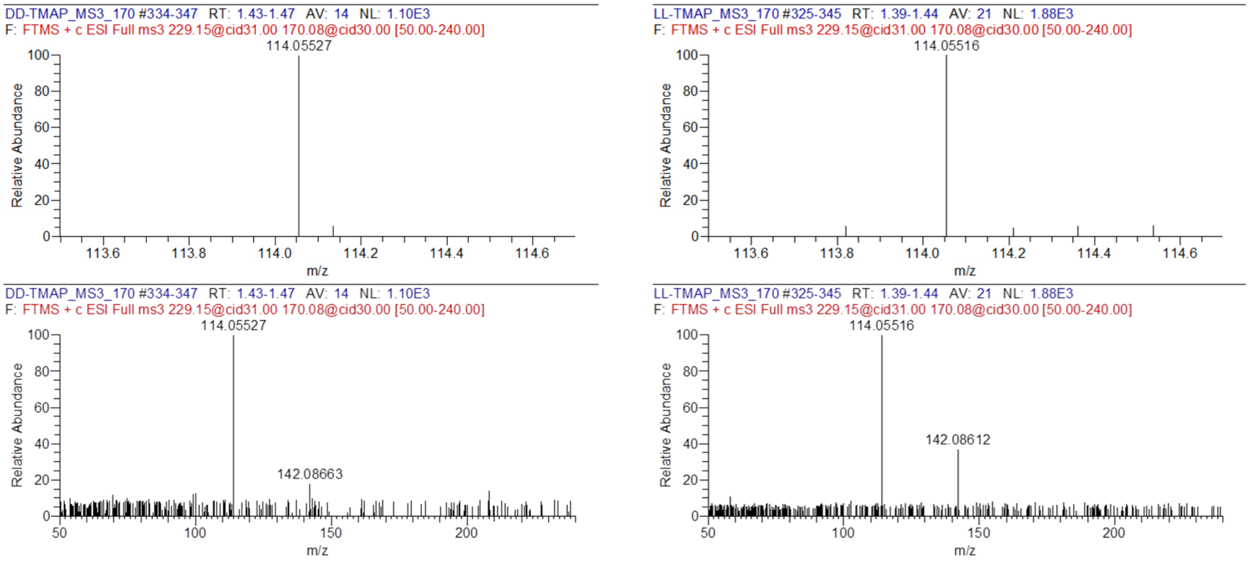

S-Figure S29. MS^3 spectra of *m/z* 229/170 of synthetic D,D-TMAP (left) and L,L-TMAP (right) with expansion (top) for comparison.

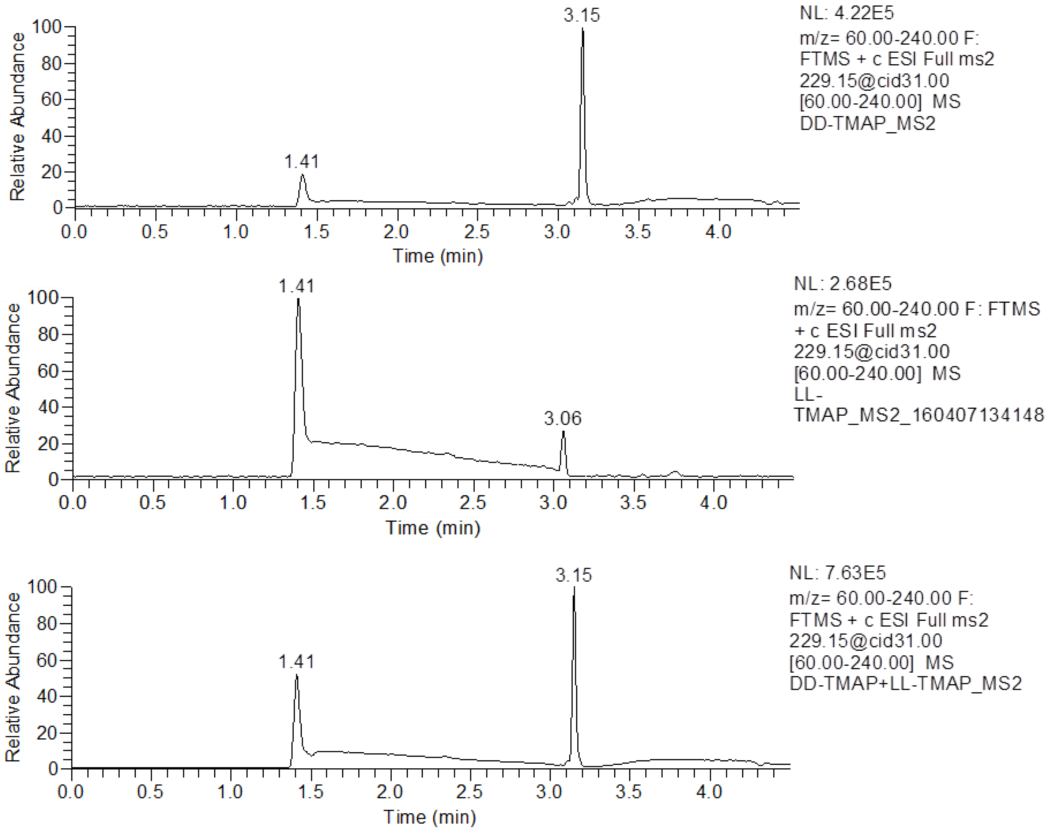

Figure S30. LC-MS/MS chromatograms of synthetic D,D-TMAP (1.41 min, top), L,L-TMAP (1.41 min, middle), and their co-injection (bottom).

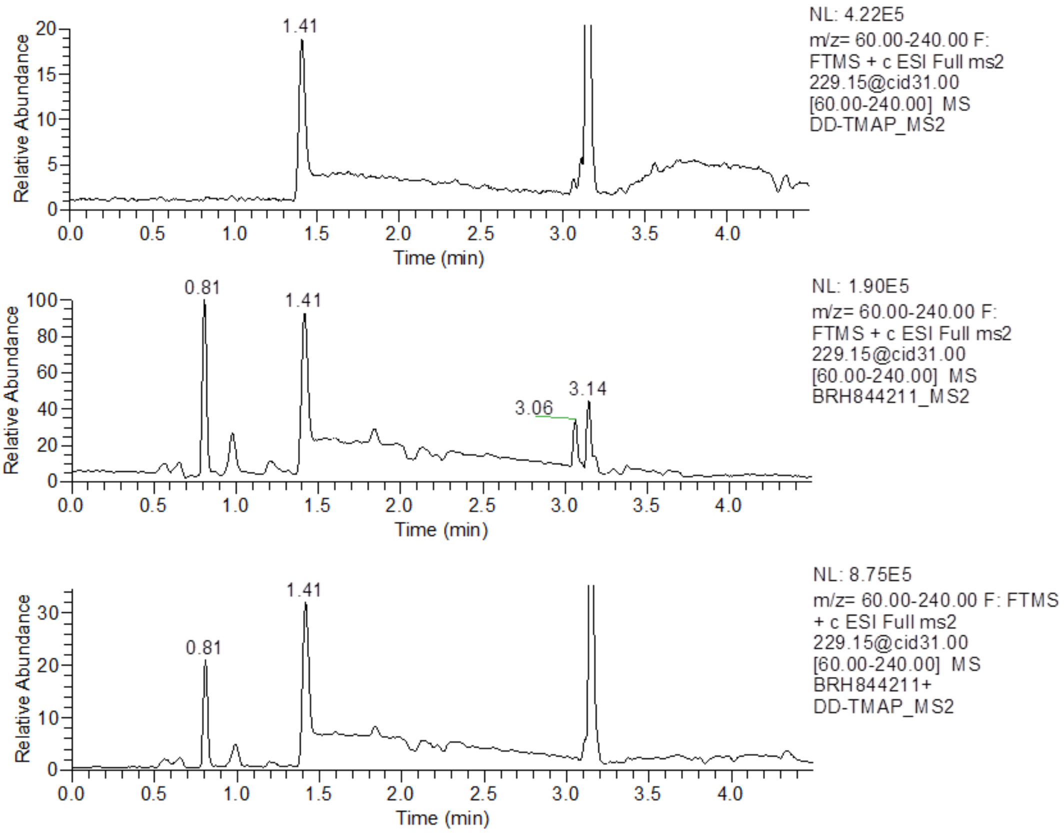

Figure S31. LC-MS/MS chromatograms of synthetic D,D-TMAP (1.41 min, top), x17299 (1.41 min, middle) in a plasma extract, and their co-injection (bottom).

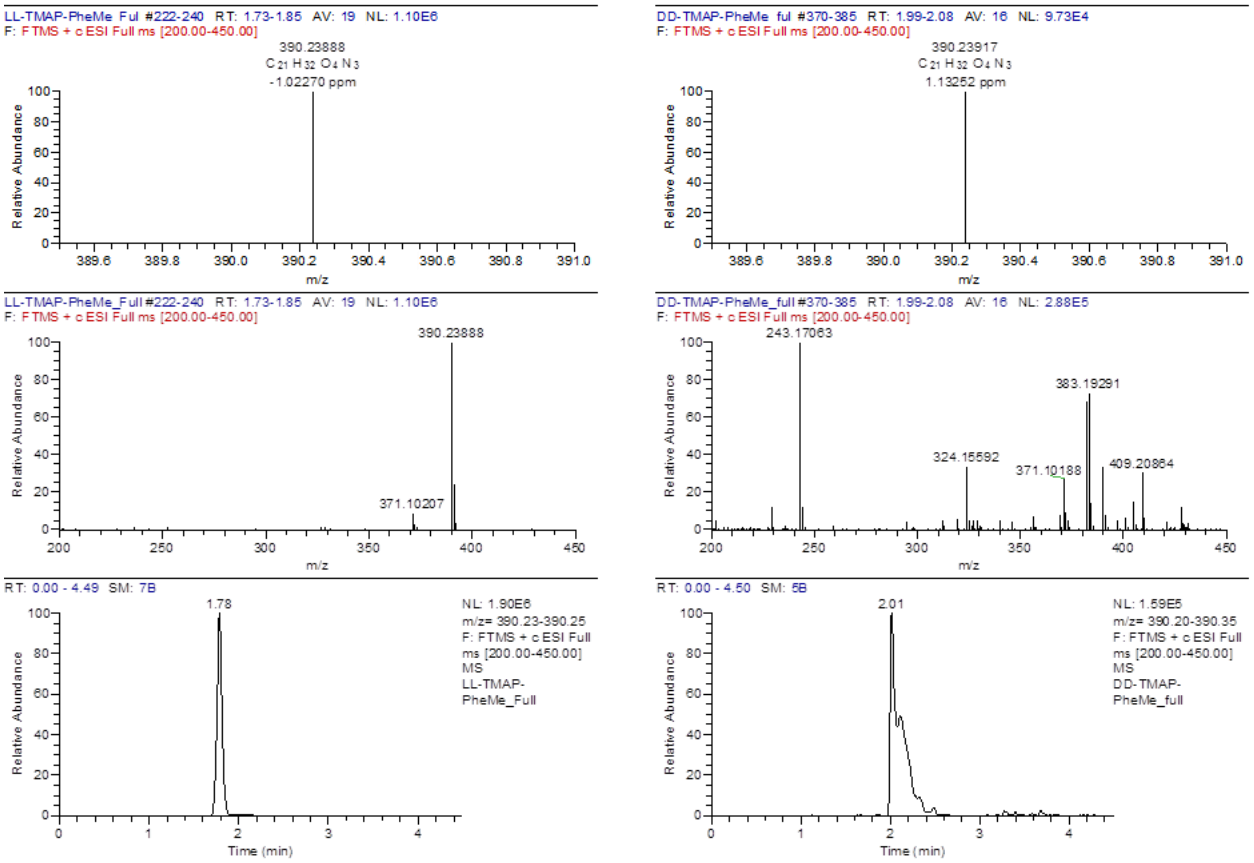

Figure S32. LC/MS chromatograms and mass spectra of L,L-TMAP-L-Phe-OMe (1.78 min, left) and D,D-TMAP-L-Phe-OMe (2.01 min, right) with expansions (top).

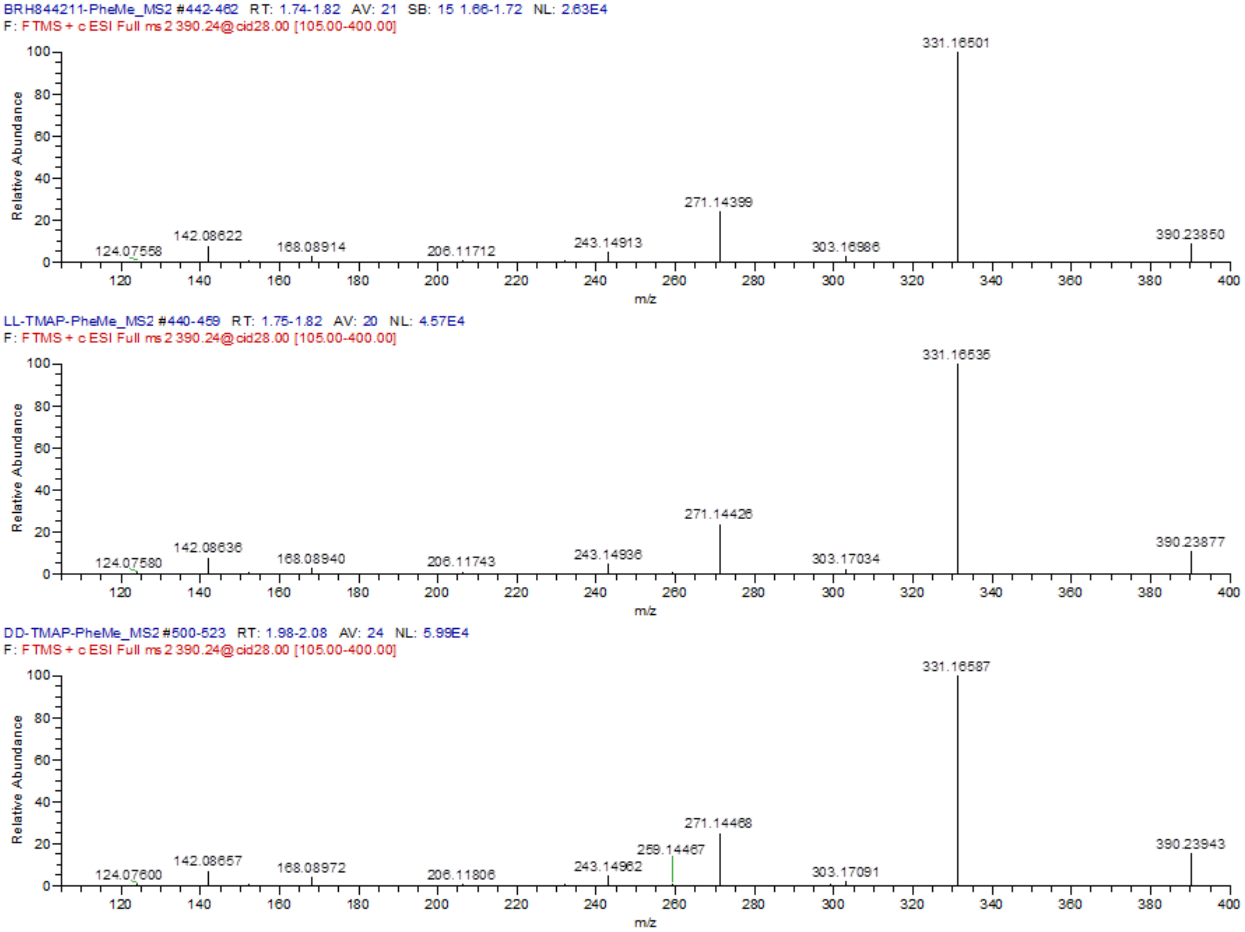

Figure S33. MS^2 spectra of L,L-TMAP-L-Phe-OMe (middle), D,D-TMAP-L-Phe-OMe (bottom), and x17299-L-Phe-OMe (top).

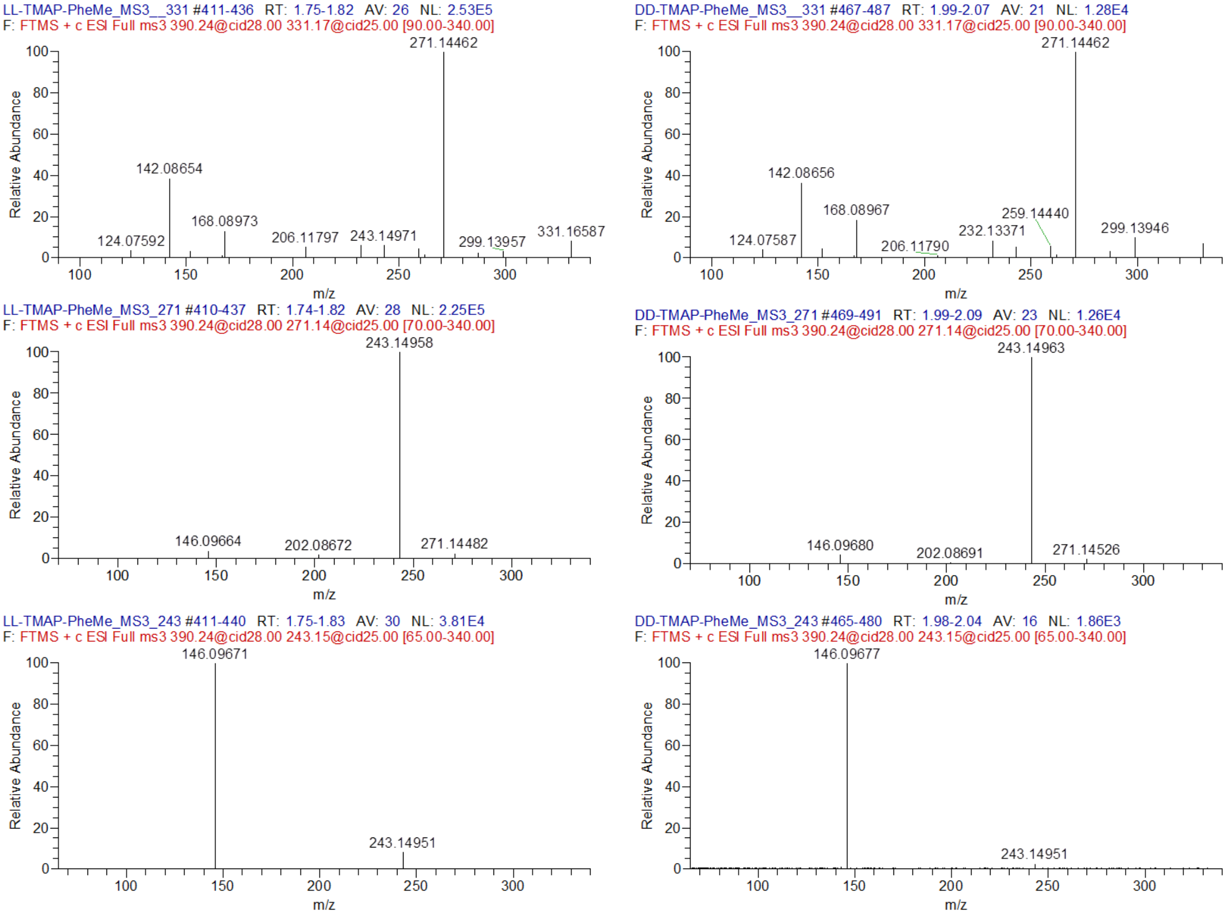

Figure S34. MS^3 spectra of *m/z* 390/331 (top), *m/z* 390/271 (middle), and *m/z* 390/243 (bottom) of L,L-TMAP-L-Phe-OMe (left) and D,D-TMAP-L-Phe-OMe (right).

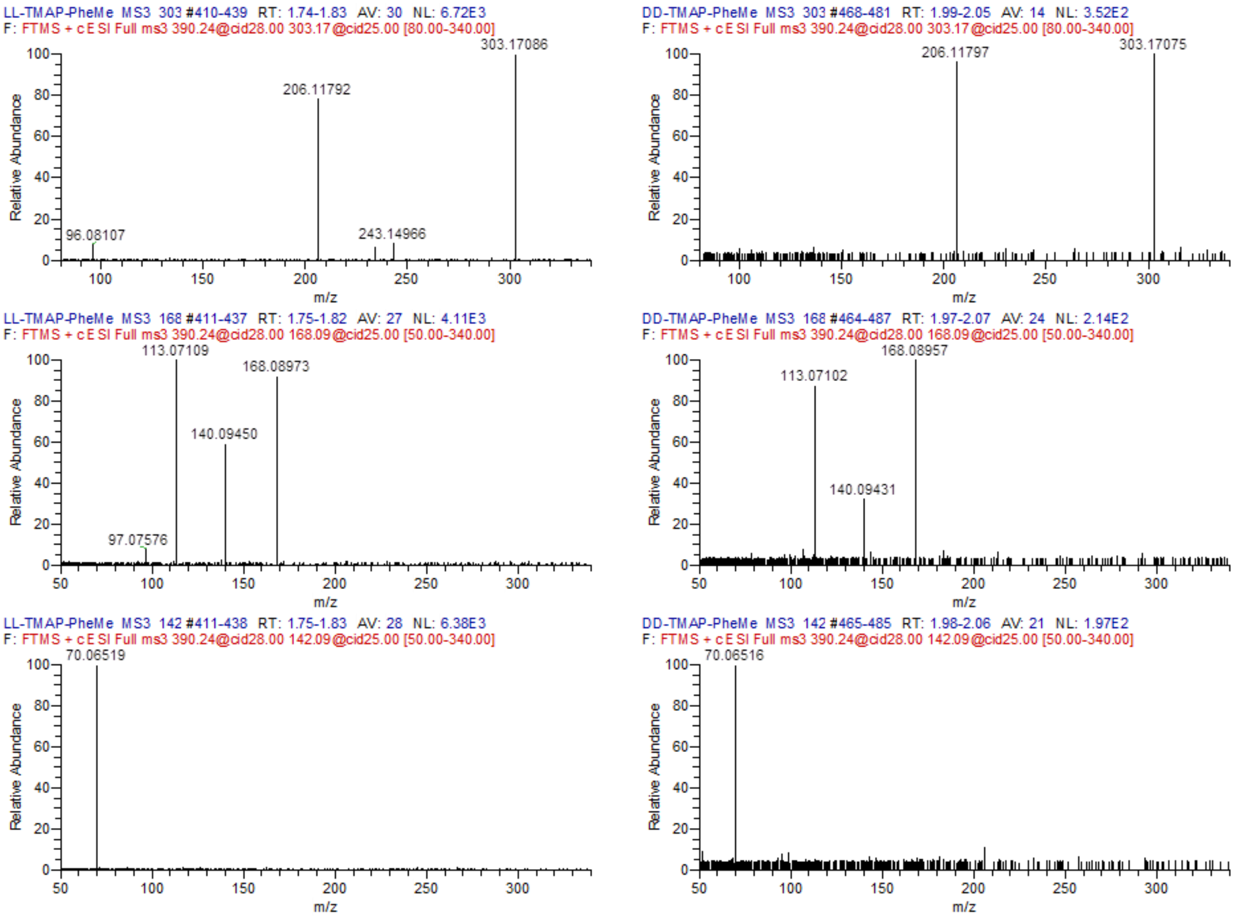

Figure S35. MS^3 spectra of *m/z* 390/303 (top), *m/z* 390/168 (middle), and *m/z* 390/142 (bottom) of L,L-TMAP-L-Phe-OMe (left) and D,D-TMAP-L-Phe-OMe (right).

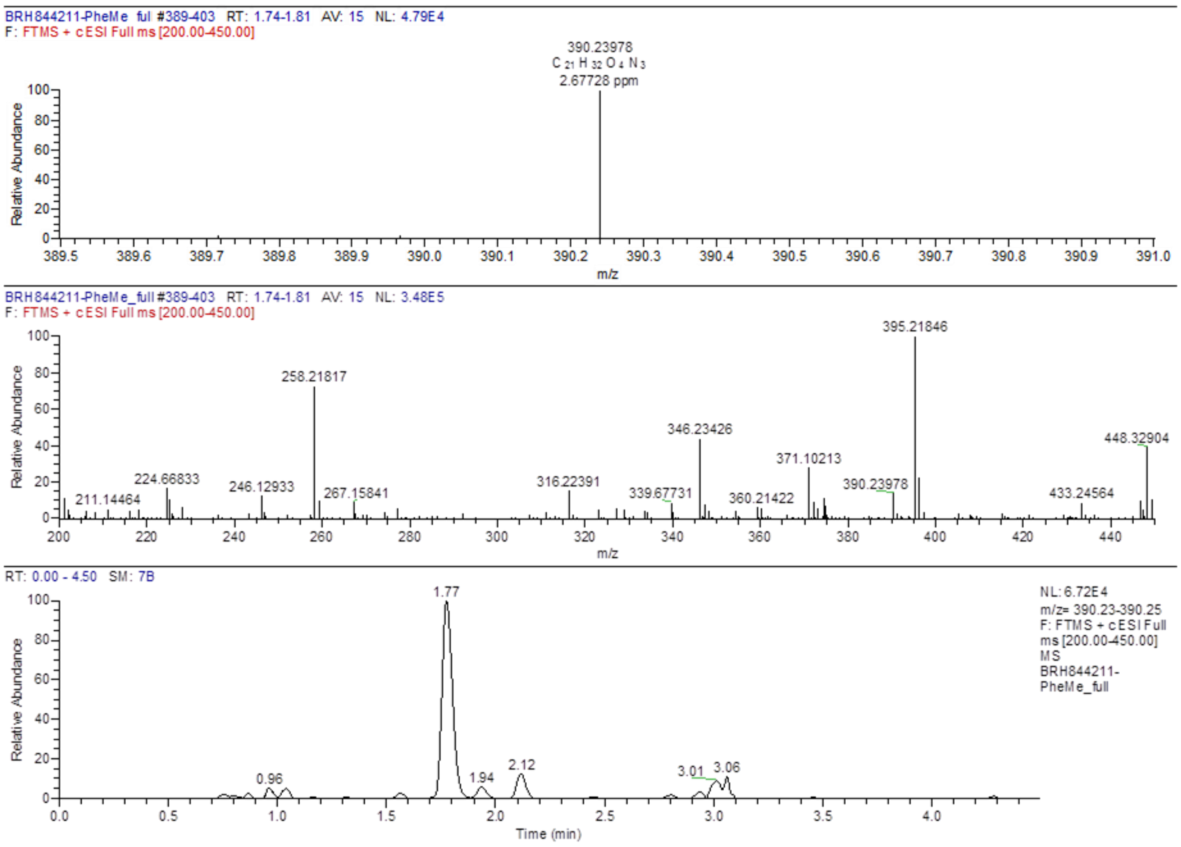

Figure S36. LC/MS chromatograms and mass spectrum of x17299-L-Phe-OMe (1.77 min) with expansion (top).

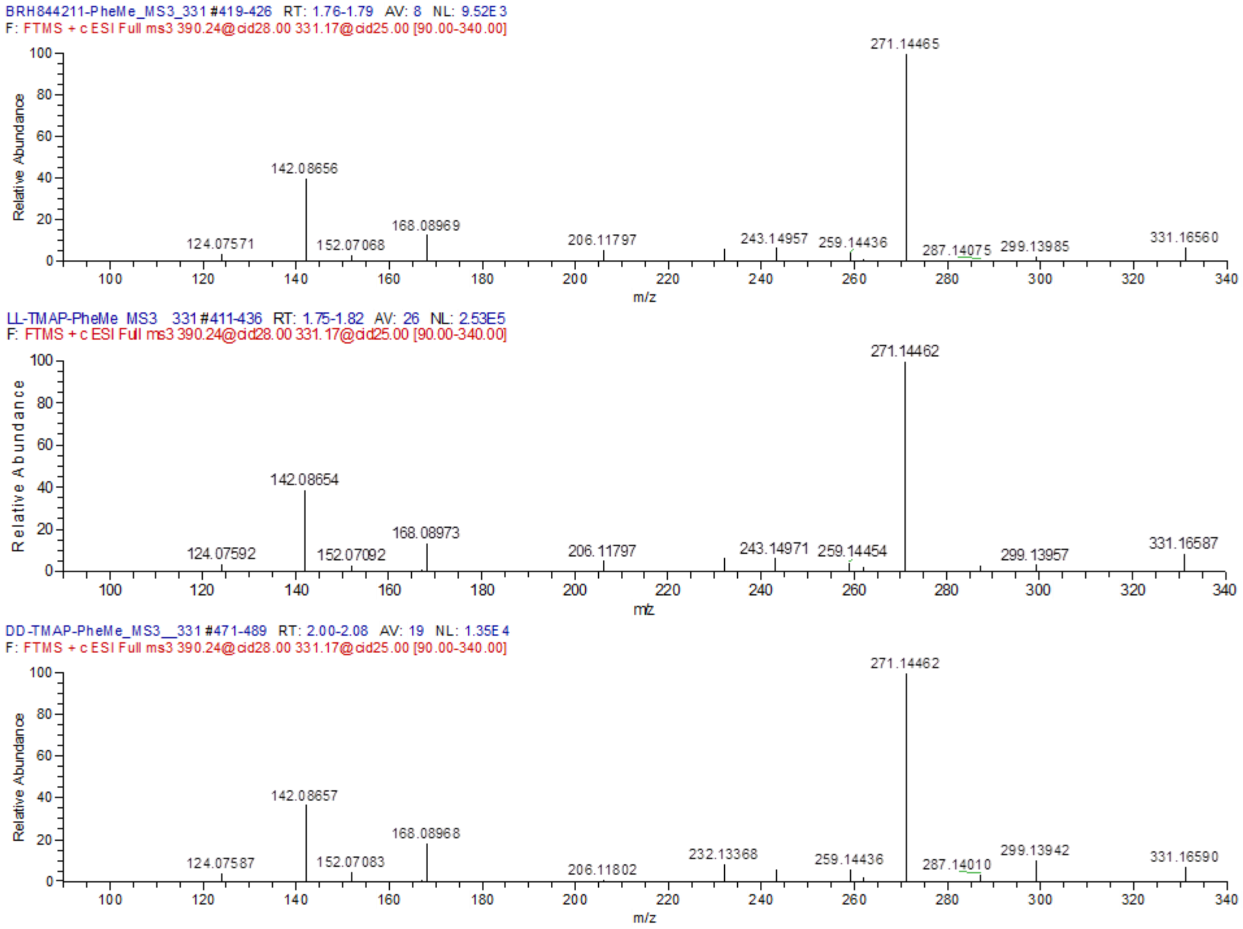

Figure S37. MS^3 spectra of *m/z* 390/331 of x17299-L-Phe-OMe (top), L,L-TMAP-L-Phe-OMe (middle), and D,D-TMAP-L-Phe-OMe (bottom).

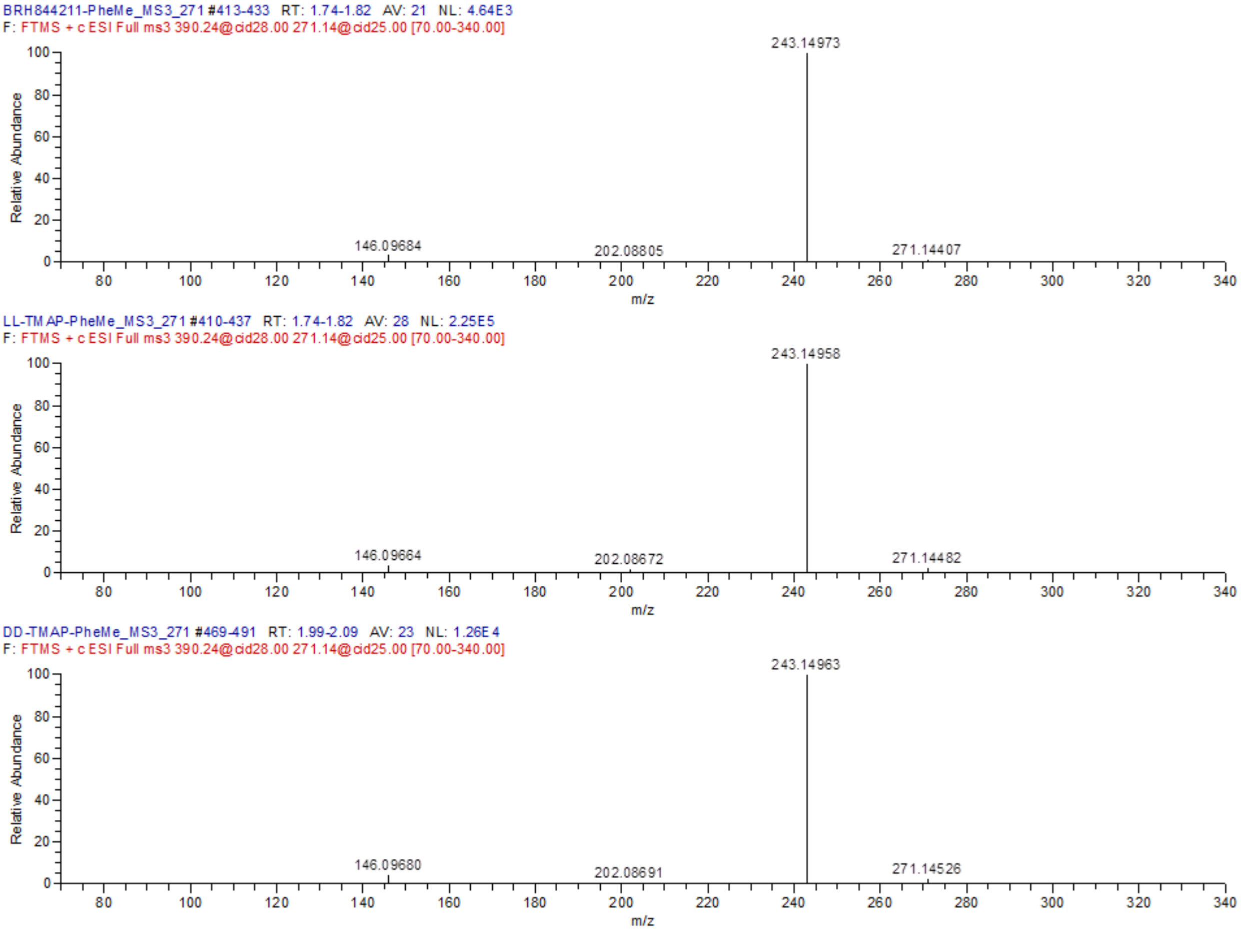

Figure S38. MS^3 spectra of *m/z* 390/271 of x17299-L-Phe-OMe (top), L,L-TMAP-L-Phe-OMe (middle), and D,D-TMAP-L-Phe-OMe (bottom).

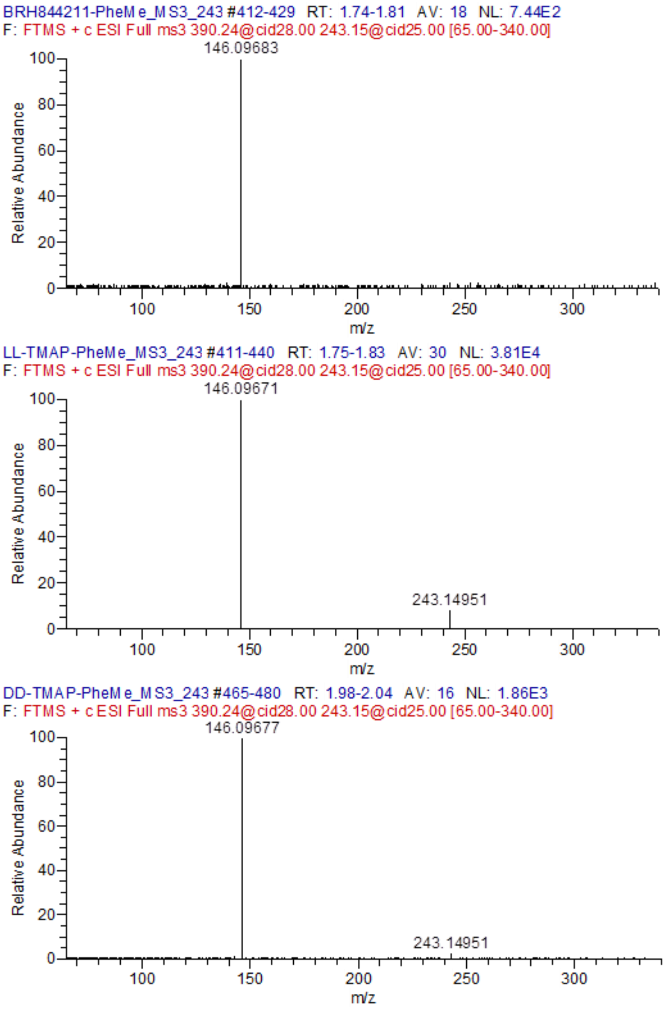

Figure S39. MS^3 spectra of *m/z* 390/243 of x17299-L-Phe-OMe (top), L,L-TMAP-L-Phe-OMe (middle), and D,D-TMAP-L-Phe-OMe (bottom).

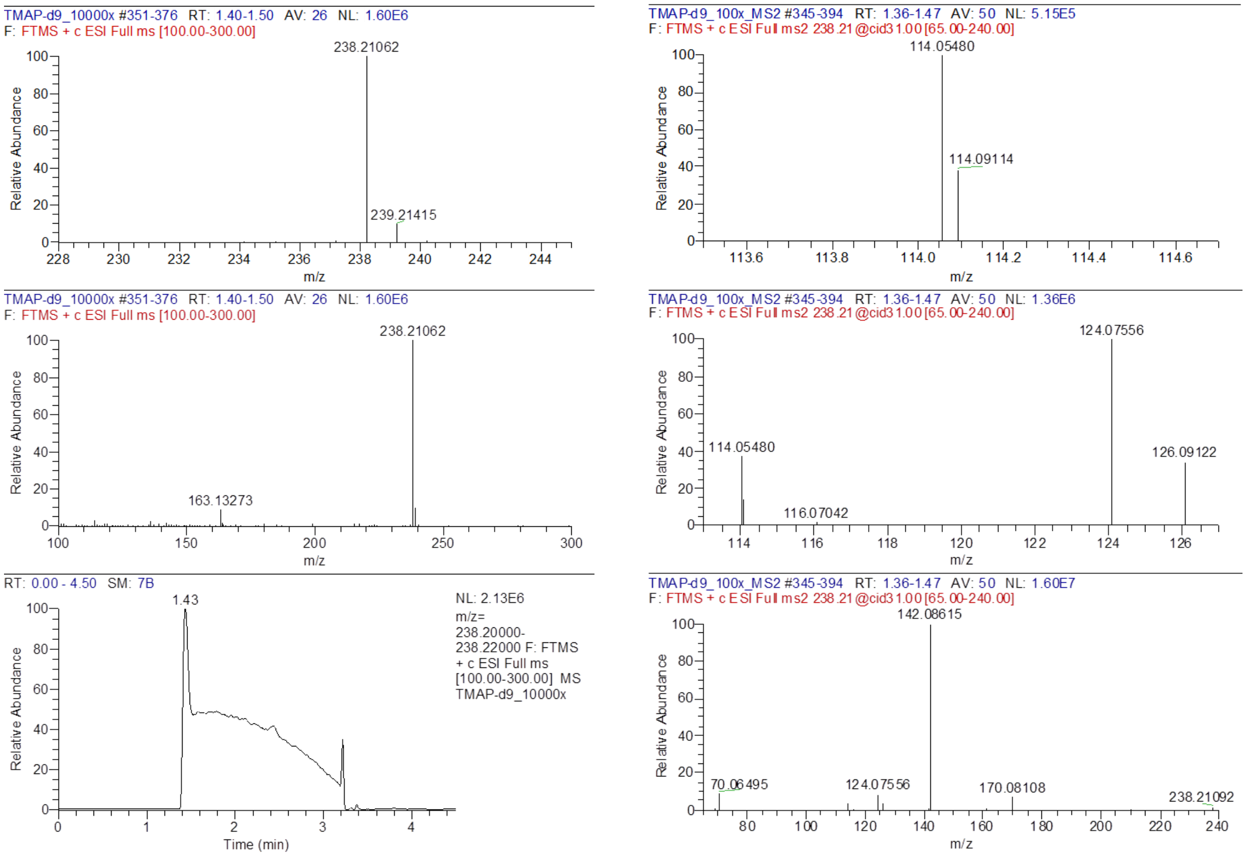

Figure S40. LC/MS chromatogram and MS spectrum (left), and MS^2 spectrum (right) of synthetic L,L-TMAP-d_9 (1.43 min) with expansions (middle and top).

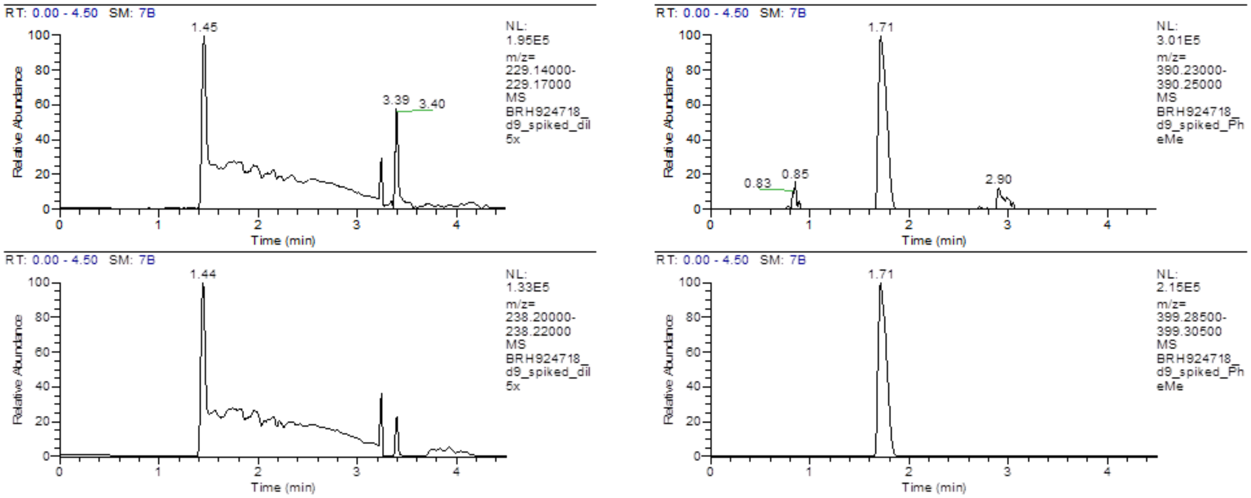

Figure S41. LC/MS chromatograms of x17299 (1.45 min, left top) and L,L-TMAP-d_9 (1.44 min, left bottom) in a spiked plasma extract, and x17299-L-Phe-OMe (1.71 min, right top) and L,L-TMAP-d_9-L-Phe-OMe (1.71 min, right bottom) in a coupling reaction mixture of the spiked plasma extract.

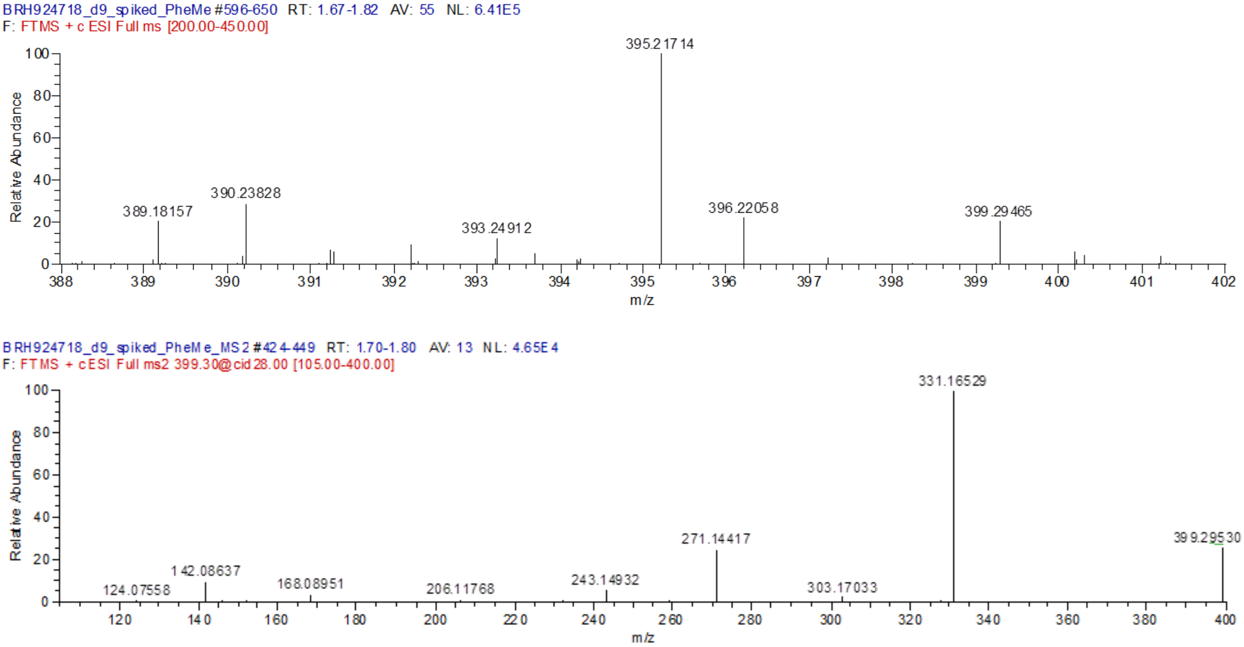

Figure S42. MS (top) and MS^2 (bottom) spectra of L,L-TMAP-d_9-L-Phe-OMe.

Scheme S1. Proposed fragmentation pathways of TMAP-L-Phe-OMe.

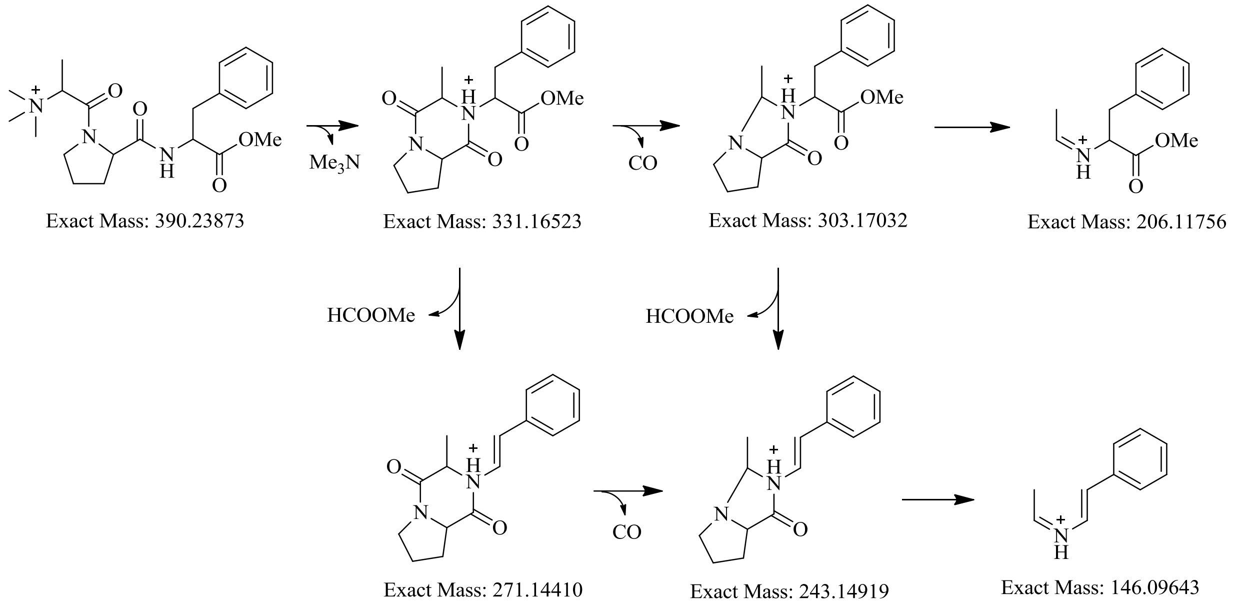

Table S1. Spectrum list of MS^2 of x17299.

FTMS + c ESI Full ms2 229.15@cid31.00 [60.00-240.00]
Scan #: 370-384
RT: 1.46-1.52
AV: 15

m/z	Intensity	Relative
70.06502	6062.9	10.74
114.05491	1535.7	2.72
114.09139	496.9	0.88
124.07568	4837.1	8.57
126.09128	1445.6	2.56
142.08628	56456.1	100.00
170.08120	4787.5	8.48
229.15460	1149.9	2.04

Table S2. Spectrum list of MS^2 of deuterated x17299.

FTMS + c ESI Full ms2 230.16@cid31.00 [60.00-240.00]
Scan #: 389-399
RT: 1.54-1.58
AV: 11

m/z	Intensity	Relative
70.06515	238.4	1.15
71.07140	2016.4	9.70
115.06133	512.3	2.47
115.09808	61.5	0.30
124.07589	1658.9	7.98
127.09769	387.7	1.87
143.09277	20781.8	100.00
171.08776	1729.7	8.32
201.94930	529.3	2.55
205.14789	161.4	0.78
219.96103	221.1	1.06
220.16469	230.3	1.11
230.16141	659.6	3.17

Table S3. Spectrum list of MS^2 of synthetic L,L-TMAP.

FTMS + c ESI Full ms2 229.15@cid31.00 [60.00-240.00]
Scan #: 350-363
RT: 1.38-1.44
AV: 14

m/z	Intensity	Relative
70.06508	16802.5	11.13
114.05503	4028.1	2.67
114.09144	1338.5	0.89
124.07580	12318.8	8.16
126.09147	3656.0	2.42
142.08642	151013.7	100.00
170.08138	12405.1	8.21
229.15488	3817.4	2.53

Table S4. Spectrum list of MS^2 of deuterated synthetic L,L-TMAP.

FTMS + c ESI Full ms2 230.16@cid31.00 [60.00-240.00]
Scan #: 372-388
RT: 1.47-1.51
AV: 17

m/z	Intensity	Relative
70.06498	6927.0	1.33
71.07128	53715.9	10.31
115.06118	13927.7	2.67
115.09750	3632.2	0.70
124.07565	43278.7	8.31
127.09761	11681.4	2.24
143.09252	520918.2	100.00
171.08743	42265.2	8.11
230.16083	18802.8	3.61

Table S5. Spectrum list of MS^2 of synthetic D,L-TMAP.

FTMS + c ESI Full ms2 229.15@cid31.00 [60.00-240.00]
Scan #: 583-615
RT: 2.31-2.41
AV: 33

m/z	Intensity	Relative
70.06505	39033.4	14.10
114.05495	6350.1	2.29
114.09137	2050.1	0.74
124.07573	29236.2	10.56
126.09139	11389.1	4.11
132.10199	3184.4	1.15
142.08635	276778.9	100.00
170.08131	81733.8	29.53
229.15483	2809.2	1.01

Table S6. Spectrum list of MS^2 of synthetic D,D-TMAP.

FTMS + c ESI Full ms2 229.15@cid31.00 [60.00-240.00]
Scan #: 351-363
RT: 1.39-1.44
AV: 13

m/z	Intensity	Relative
70.06512	4449.0	10.21
114.05496	1077.3	2.47
114.09155	296.5	0.68
124.07582	3489.2	8.01
126.09151	983.4	2.26
142.08648	43558.4	100.00
170.08140	3615.5	8.30
229.15533	1044.3	2.40

Table S7. Spectrum list of MS^2 of L,L-TMAP-L-Phe-OMe.

FTMS + c ESI Full ms2 390.24@cid28.00 [105.00-400.00]
Scan #: 440-459
RT: 1.75-1.82
AV: 20

m/z	Intensity	Relative
124.07580	346.0	0.76
142.08636	3664.1	8.01
152.07061	296.2	0.65
168.08940	1324.5	2.90
206.11743	485.4	1.06
232.13357	292.5	0.64
243.14936	2279.7	4.99
259.14427	321.0	0.70
271.14426	10861.6	23.75
303.17034	1104.7	2.42
331.16535	45726.0	100.00
390.23877	4967.2	10.86

Table S8. Spectrum list of MS^2 of D,D-TMAP-L-Phe-OMe.

FTMS + c ESI Full ms2 390.24@cid28.00 [105.00-400.00]
Scan #: 500-523
RT: 1.98-2.08
AV: 24

m/z	Intensity	Relative
124.07600	577.3	0.96
142.08657	4296.1	7.17
152.07107	540.9	0.90
168.08972	2505.3	4.18
206.11806	394.6	0.66
232.13390	662.1	1.11
243.14962	2857.4	4.77
259.14467	741.8	1.24
271.14468	15148.8	25.29
299.13955	817.1	1.36
303.17091	1296.1	2.16
331.16587	59895.0	100.00
390.23943	9307.6	15.54

Table S9. Spectrum list of MS^2 of x17299-L-Phe-OMe.

FTMS + c ESI Full ms2 390.24@cid28.00 [105.00-400.00]
Scan #: 442-462
RT: 1.74-1.82
AV: 21

m/z	Intensity	Relative
124.07558	166.0	0.63
142.08622	2102.2	8.00
152.07030	170.2	0.65
168.08914	768.8	2.93
206.11712	240.3	0.91
232.13340	197.2	0.75
243.14913	1296.6	4.93
259.14347	141.6	0.54
271.14399	6378.1	24.27
277.15486	157.8	0.60
303.16986	660.6	2.51
319.18594	523.0	1.99
331.16501	26278.2	100.00
390.23850	2336.8	8.89

Table S10. Spectrum list of MS^2 of synthetic L,L-TMAP-d_9.

FTMS + c ESI Full ms2 238.21@cid31.00 [65.00-240.00]
Scan #: 345-394
RT: 1.36-1.47
AV: 50

m/z	Intensity	Relative
70.06495	1479674.6	9.23
114.05480	514823.2	3.21
114.09114	198927.7	1.24
124.07556	1363974.4	8.51
126.09122	468917.8	2.93
142.08615	16028258.0	100.00
170.08108	1176932.0	7.34
238.21092	296029.2	1.85

Table S11. Spectrum list of MS^2 of L,L-TMAP-d_9-L-Phe-OMe.

FTMS + c ESI Full ms2 399.30@cid28.00 [105.00-400.00]
Scan #: 424-448
RT: 1.70-1.80
AV: 13

m/z	Intensity	Relative
124.07558	423.3	0.91
142.08637	4517.1	9.72
152.07072	279.2	0.60
168.08951	1722.6	3.71
206.11768	529.7	1.14
232.13337	207.5	0.45
243.14932	2729.3	5.87
259.14395	352.0	0.76
271.14417	11416.8	24.56
303.17033	1152.3	2.48
328.13257	417.7	0.90
331.16529	46483.5	100.00
399.29530	12117.9	26.07
